# Unlocking the potential of battery technologies through Time-of-Flight Secondary Ion Mass Spectrometry

**DOI:** 10.1080/14686996.2025.2580917

**Published:** 2025-12-09

**Authors:** Prince Sharma, Gen Hasegawa, Sihao Xing, Naoaki Kuwata

**Affiliations:** a Center for Green Research on Energy and Environmental Materials, National Institute for Materials Science (NIMS), Tsukuba, Japan; b Graduate School of Chemical Sciences and Engineering, Hokkaido University, Sapporo, Japan

**Keywords:** TOF-SIMS, battery, degradation, interphases, AI integration

## Abstract

In response to the evolving demands in energy storage, the review underscores the critical need for ongoing research in battery technology, specifically centred to indispensable role of Time-of-Flight Secondary Ion Mass Spectrometry (TOF-SIMS) which aims to characterize the intricate components of all kind of batteries. This article extensively examines SIMS applications, illuminating aspects such as chemical compositions, structural arrangements, electronic behaviours, and various parameters like bulk and grain diffusion coefficients. All these effective parameters analysis extends to the discussion of in-situ studies, suggesting the potential for operando SIMS and emphasizing their vital role in real-time monitoring during battery operations. These studies unveil intricate interactions at solid-solid interfaces, exerting a significant influence on overall battery performance. By spotlighting recent advances and emerging trends, the review summarizes and examines case studies by various researchers on the use of SIMS for analysing battery-related materials.

## IMPACT STATEMENT

This review uniquely summarizes four decades of TOF-SIMS in battery research, highlighting its evolving role from interfacial analysis to operando studies and integration with complementary characterization techniques.

## GRAPHICAL ABSTRACT

**Figure:**
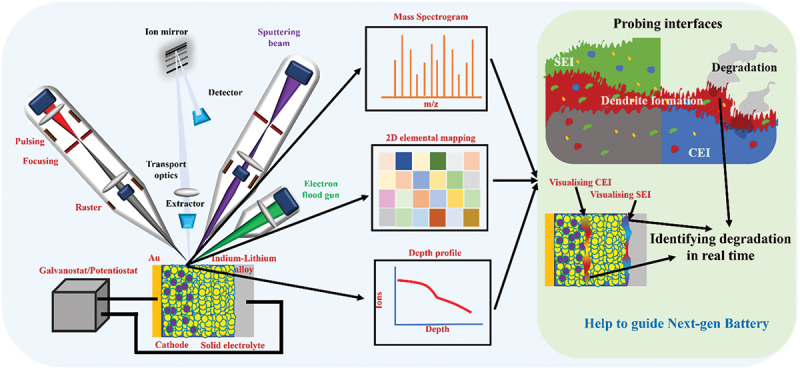

## 1. Introduction

The evolution of batteries is a compelling exploration marked by significant discoveries and technological milestones that have reshaped their trajectory over the years [1–5]. In the 19th century, Michael Faraday established the foundation for future developments in solid-state Ionics by conducting groundbreaking research on solid electrolytes [6]. Advancements in the late 1950s saw the emergence of silver-conducting electrochemical systems utilizing solid electrolytes [7]. However, challenges such as low energy density persisted. In 1967, a transformative moment occurred in β - alumina with the discovery of fast ionic conduction, sparking enthusiasm and driving the expansion of novel electrochemical devices [8]. Early innovations, such as molten sodium/β - alumina/sulphur cells, exemplified the pioneering efforts of entities like Ford Motor Company and NGK [8,9]. Though, 1990s brought forth challenges, with many systems requiring elevated temperatures. A breakthrough in the form of lithium – phosphorus oxynitride (LiPON) emerged, paving the way for thin-film Lithium ion batteries (LIB) [10]. However, challenges such as thin-film electrolyte deposition costs persisted. A paradigm shift occurred with the study by Kamaya et al. in 2011, where they demonstrated a solid electrolyte Li_10_GeP_2_S_12_, which boasts bulk ionic conductivity surpassing its counterparts liquid electrolyte at room temperature [11]. This breakthrough indicated a starting of new era, where solid-ionic materials enabling technologically compete with counterparts of Li-ion.

The millennium era witnessed a renewed surge of interest in solid-state battery (SSB) technologies, driven primarily by advancements in the automotive and transportation industries. Collaborations between major companies like Bolloré, Toyota, and Volkswagen underscored the industry’s commitment. In 2013, the University of Colorado Boulder introduced a solid-state lithium battery featuring a solid composite cathode based on iron – sulphur chemistry, offering the potential for significantly increased energy capacity [12]. In 2017, John Goodenough introduced a solid-state glass battery incorporating a glass electrolyte and an alkali-metal anode, marking a significant advancement in battery technology [13]. Technological strides continued with startups such as Solid Power and QuantumScape securing substantial investments in 2018 [14]. The automotive industry played a pivotal role, with key players like Toyota, Ford, and BMW funding initiatives and advancing the cause of SSBs technologies. Toyota deepened its partnership with Panasonic, emphasizing the pursuit for more efficient and safer energy storage solutions in 2020 [15]. Toyota, in 2021, announced plans to incorporate SSBs into future car models, beginning with hybrid models in 2025 [16]. Ford and BMW jointly invested $150 million in Solid Power, reflecting collaborative efforts to propel SSB technologies forward [16]. In 2022, Svolt announced the development of a high-density electric battery, while Swiss Clean Battery outlined plans to build the world’s first sustainable SSB factory [17,18]. Additionally, ProLogium entered a technical cooperation agreement with Mercedes-Benz, reflecting the increasing collaborative initiatives within the battery industry [19]. As the trajectory of SSBs unfolds, Maxell Corporation achieved a significant milestone in June 2023, initiating mass production of large-capacity SSBs [20]. These developments underscore the dynamic landscape and collaborative spirit driving advancements in SSBs technologies globally.

Upon closer examination of the challenges posed by battery technology, researchers are actively addressing a range of complex issues. These challenges encompass significant manufacturing costs associated with thin-film SSBs, difficulties in low-temperature and high-pressure operations affecting overall efficiency, and interfacial resistance between cathodes and solid electrolytes, which is crucial for efficient energy transfer at this interface [3,21,22]. Additionally, factors such as grain boundary defects and variability in diffusion coefficients at different contact edges further contribute to the intricacies of the technology [3,22]. While the initial challenges related to manufacturing, temperature, and pressure sensitivity are well-known in SSBs. Despite, the effects of grain boundaries, variable diffusion coefficients at different cathode sites, and interfacial resistance become apparent only through visualization techniques, especially using spectroscopy, such as Time-of-Flight Secondary Ion Mass Spectrometry (TOF-SIMS) [23–27]. The importance of spectroscopic techniques in the realm of SSBs cannot be overstated, as these methods play a pivotal role in unravelling the intricate complexities of battery materials. Spectroscopy, encompassing various techniques such as Raman Spectroscopy [28–31], X-ray Photoelectron Spectroscopy (XPS) [32–36], Fourier Transform Infrared (FTIR) Spectroscopy [37–40], Nuclear Magnetic Resonance (NMR) [41–44], UV-Visible-Near-Infrared Spectroscopy [30,45–48], and SIMS [49–57], serves as an invaluable toolbox for researchers aiming to address key research questions and overcome challenges in SSBs. One of the fundamental contributions of spectroscopy lies in its ability to provide insights into the microstructural, interfacial, chemical, and electronic properties along with oxidative and reduction outcomes of SSB materials. Researchers leverage these techniques to examine the composition of complex components, including solid electrolytes, cathodes, and anodes. Spectroscopy facilitates a deep understanding of how these materials evolve during various electrochemical processes, offering crucial information for the optimization of battery performance.

Spectroscopic techniques play a crucial role in addressing challenges associated with SSBs by offering customized solutions. Specifically, the issue of interfacial resistance between cathodes and solid electrolytes can be alleviated through thorough spectroscopic analysis. This allows researchers to fine-tune the design and composition of these crucial interfaces for enhanced performance. One key spectroscopic technique that proves valuable in this context is TOF-SIMS [3,26,58–62]. TOF-SIMS is a dominant experimental variant of molecular SIMS that provides higher mass resolution, allowing for more precise identification of ions [58,63]. TOF-SIMS is particularly valuable in surface analysis applications where detailed information about the molecular composition of the top few nanometres of a sample is crucial. It is often employed in areas like biomaterials, polymers, and organic coatings. It also contributes to monitoring and comprehending the elemental and molecular composition of various battery components, such as the cathode, anode, electrolyte, and separator. It enables the examination of element and molecule distribution within the battery, shedding light on their impact on performance. It also facilitates interface analysis which involves studying the chemical composition and structure of interfaces to understand their influence on processes like charge transfer and ion transport. Moreover, given that battery failures can stem from factors like material degradation, manufacturing defects, or improper operation, SIMS becomes instrumental in analysing materials and identifying the root cause of failures. In summary, SIMS serve as indispensable tools for researchers navigating the complexities of all kind of batteries. By providing detailed information on material properties, facilitating in-depth analysis of interfaces, and offering real-time insights into electrochemical processes, SIMS not only addresses key research questions but also holds the key to resolving challenges, paving the way for the advancement of safe, high-energy-density, and enduring all kind of batteries. Moreover, the evolution of in situ or operando SIMS has revolutionized the field by enabling real-time monitoring of electrochemical processes during battery operation. Thus, the integration of advanced spectroscopic techniques, notably TOF-SIMS, not only addresses current challenges in all kind of batteries but also propels the development of more robust and enduring battery materials, paving the way for a future of enhanced energy storage technologies.

## 2. TOF-SIMS principles and applications

In the realm of surface analysis and depth profiling, TOF-SIMS has emerged as a compelling technique, bringing forth several advantages. SIMS is vital for monitoring the elemental and molecular composition of battery components, like the cathode, anode, electrolyte, and separator. It examines element and molecule distribution, revealing their impact on performance and facilitating interface analysis for understanding processes like charge transfer and ion transport. Moreover, SIMS is instrumental in analysing materials to identify the root causes of battery failures, addressing issues like material degradation, manufacturing defects, or improper operation. However, achieving an accurate interpretation of TOF-SIMS data requires careful consideration, too of various factors and limitations. Therefore, a comprehensive understanding of the working principle of TOF-SIMS, along with its primary advantages and drawbacks, is essential. This section delves into these crucial aspects. Furthermore, information about the main instrument providers and setups is presented.

A number of established companies supply Secondary-Ion Mass Spectrometry (SIMS) instruments across TOF-SIMS, magnetic-sector SIMS, quadrupole SIMS, NanoSIMS, and FIB-SIMS platforms. Key manufacturers include IONTOF GmbH and ULVAC-PHI for advanced TOF-SIMS systems; Hiden Analytical for quadrupole-based SIMS; and Thermo Fisher Scientific for FIB-integrated TOF-SIMS solutions. High-precision ion-microprobe instruments are produced by CAMECA, known for the NanoSIMS and IMS series, and Australian Scientific Instruments, which develops the SHRIMP microprobes. Specialist or compact builders such as Kore Technology and Toyama Engineering contribute modular TOF-SIMS and custom FIB-TOF-SIMS systems. Additional companies often listed in market reports, including Rigaku, JEOL, Shimadzu, and TOFWERK, offer TOF analyzers or hybrid SIMS-compatible platforms.

### 2.1. History of TOF-SIMS

TOF-SIMS is a mass spectrometric technique in which a pulsed primary ion beam bombards the sample surface, and the resulting secondary ions are accelerated into a field-free drift region. Their time of flight to the detector is used to determine the mass-to-charge ratio (*m/z*) with high sensitivity and rapid acquisition [57,58,63]. TOF-SIMS emerged as a dominant experimental variant of molecular SIMS due to its parallel detection of all masses, enabling efficient acquisition of high-resolution chemical maps over large fields of view.

Historically, SIMS development began in the early 1970s with two operational regimes: dynamic SIMS, employing a high current density ion gun that achieved deep profiling but at the cost of sample damage, and static SIMS (S-SIMS), pioneered by Benninghoven, which introduced a low primary ion current for probing only the upper monolayers of surfaces [63,64]. In conventional SIMS, the magnetic sector analyser was the standard choice, offering very high mass resolution (M/ΔM > 10,000) and, in recent systems, spatial resolution down to ~50 nm. These strengths make sector SIMS especially effective for isotope analysis and depth profiling with quantitative accuracy, and its performance continues to be valuable in specialized fields such as semiconductor doping studies and fundamental isotope geochemistry [63,65]. However, sector SIMS instruments face limitations in terms of transmission efficiency and sequential mass scanning, which restrict their applicability for complex, heterogeneous systems. While Quadrupole SIMS offered faster scanning but only detected a single *m/z* at a time, reducing throughput and sensitivity compared to TOF analysers. The introduction of TOF analysers in the late 1980s transformed SIMS, enabling simultaneous detection across the full mass range (up to m/z ~ 10,000) with higher ion transmission, improved sensitivity for molecular fragments, and rapid chemical imaging [63,65]. Advancements such as time-to-digital conversion, ion mirrors, and microchannel plate detectors further enhanced both mass accuracy and spatial mapping capabilities.

In the context of battery research, TOF-SIMS demonstrates clear superiority. Its ability to acquire high-sensitivity, three-dimensional chemical maps allows for nanoscale visualization of lithium, sodium, and sulfur distributions in electrodes and electrolytes, detection of solid – electrolyte interphases (SEI), and monitoring of degradation products at buried interfaces. While sector SIMS excels in ultimate mass and spatial resolution for selected applications, TOF-SIMS provides the combination of speed, molecular sensitivity, and 3D chemical imaging necessary for studying complex, heterogeneous, and reactive electrochemical materials. Recent developments such as focused ion beam (FIB) SIMS [66–71] and fluorine gas-supplied SIMS [50], as well as multivariate analysis techniques like principal component analysis (PCA) [72], have further expanded the role of TOF-SIMS in addressing pressing questions in energy storage materials.

### 2.2. Basic principle and working of TOF-SIMS

**Figure 1.**
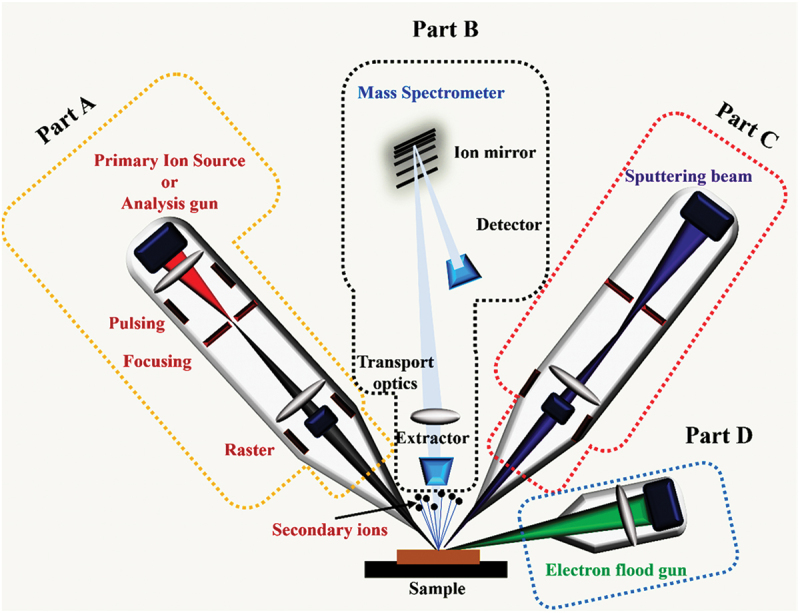
Schematic of Time-of-Flight Secondary Ion Mass Spectrometry (TOF-SIMS) setup (inspired from ION-TOF instrument at National Institute of Materials Science, Japan).

The operational mechanism of SIMS involves a series of intricate steps, commencing with the bombardment of the sample surface by a primary ion beam, leading to the ejection of ions in which mainly the secondary ions are detected and analysed at the spectrometer. It provides a mass spectrum of the surface that enables in-depth chemical analysis. Each element of this process is outlined below, as shown in Figure 1:

- i. Central to SIMS is the primary ion beam source (Part A, Figure 1), typically a Liquid metal ion gun (LMIG) – Ga^+^, Bi_*x*_^+^. Utilizing Bi_*x*_^+^ (monoatomic or clusters) as the primary ion species, the primary ion beam is accelerated to bombard the sample surface in ultrahigh vacuum conditions (≤10^−8^ mbar) an eject ions [63,65]. It is crucial to emphasize that the total ion dose in S-SIMS must adhere to a defined limit. This limit is expressed as the product of the primary ion flux or current density and the total analysis time (*t*), and it should satisfy the inequality:
  (1) $$\begin{array}{c} \frac{I_{p}}{\mathrm{ze}}t\leq \frac{1}{\delta }A \end{array}$$

    Here *I*_*p*_ represents the primary ion current, *δ* is the damage cross section, *A* is sample area and *ze* is charge of the primary ions. Typically, primary ion current densities in the range of µA cm^−2^ and nA cm^−2^ are employed in dynamic and S-SIMS, respectively. Furthermore, in TOF-SIMS, the choice of primary ion current density spans a wide range, depending on the mode of analysis. Adherence to these specified limits is crucial to control and mitigate sample damage during analysis.

ii. The secondary ions provide a snapshot of the surface’s elemental and isotopic composition, including atomic, electrons, small clusters ions, and molecular fragments, typically from the first monolayers of the sample [63,65]. The ion mixing effect, influenced by material hardness, can influence the depth of information. Although the use of large cluster ions as primary ion species usually reduces surface sensitivity, it offers advantages such as decreased implantation depth and less fragmentation of molecular samples.

iii. Finally, the generated secondary ions are then accelerated into a mass spectrometer (Part B, Figure 1) for analysis. The mass spectrometer, crucial for separating and measuring mass-to-charge ratios, achieves this by applying electric fields, directing ions along distinct trajectories based on their mass-to-charge ratios. In TOF-SIMS, the mass-to-charge ratio (m/z) of each ion is determined by accelerating them through an electric field, measuring the time taken to reach the detector after traversing a field-free flight tube [63,65]. The resulting mass spectrum graphically represents the abundance of ions at various mass-to-charge ratios, with each peak corresponding to a specific element or isotope. Researchers analyse these peaks and intensities to derive valuable insights into the elemental composition, isotopic ratios, and molecular structure of the sample.

iv. In depth profiling mode, a sputtering ion beam (Part C, Figure 1), typically operating at low energy and high current, is used to sequentially remove surface layers without generating analytical signals. Common sputtering species include Cs^+^, which enhances detection of electronegative ions (e.g. O^−^, Cl^−^); O_2_^+^/O^−^, which improves ionization of electropositive elements (e.g. Na^+^, K^+^, Fe^+^); Ar^+^, used for inert, non-reactive sputtering; and Ar cluster ions (Ar_*n*_^+^) or gas cluster ion beams (GCIB), which enable low-damage sputtering of organic and soft materials.

v. It is important to note that during SIMS analysis, the sample surface can sometimes accumulate charge due to the continuous bombardment of primary ions, particularly when analysing insulating materials. This surface charging can distort the trajectories of the emitted secondary ions, leading to inaccurate mass spectra and degraded spatial resolution. To counteract this effect and maintain the accuracy of the analysis, a charge compensation system, commonly known as a flood gun (Part D, Figure 1), is employed. The flood gun emits a stream of low-energy electrons (or occasionally ions) to neutralize the positive charge buildup on the sample surface, thereby stabilizing the measurement conditions and ensuring reliable SIMS performance.

SIMS is sensitive to surface contamination, necessitating precautions to minimize or account for external factors that might influence results. Calibration using standards with known compositions is a common practice to ensure data accuracy and reliability [63,65]. It’s crucial to acknowledge that TOF-SIMS is a destructive method that utilizes fragmentation information to infer surface chemistry. Detected secondary ions represent fragments of existing compounds formed during the collision cascade and associated effects, such as material recombination/mixing. Despite its semi-quantitative nature due to unknown ionization probabilities, normalization procedures and calibration curves using model samples contribute to meaningful conclusions. The linear relationship between secondary ions current and ionization probability presents both advantages and challenges, offering high sensitivity for compounds with high ionization probability but complicating precise quantitative evaluation [63,65]. TOF-SIMS can be integrated with features like a sputter gun or focused-ion beam for depth profiling and 3D tomography analysis, enhancing its versatility for surface-sensitive and bulk analyses [58].

### 2.3. Modes of operation (surface imaging, surface spectroscopy, and depth profiling)

**Figure 2.**
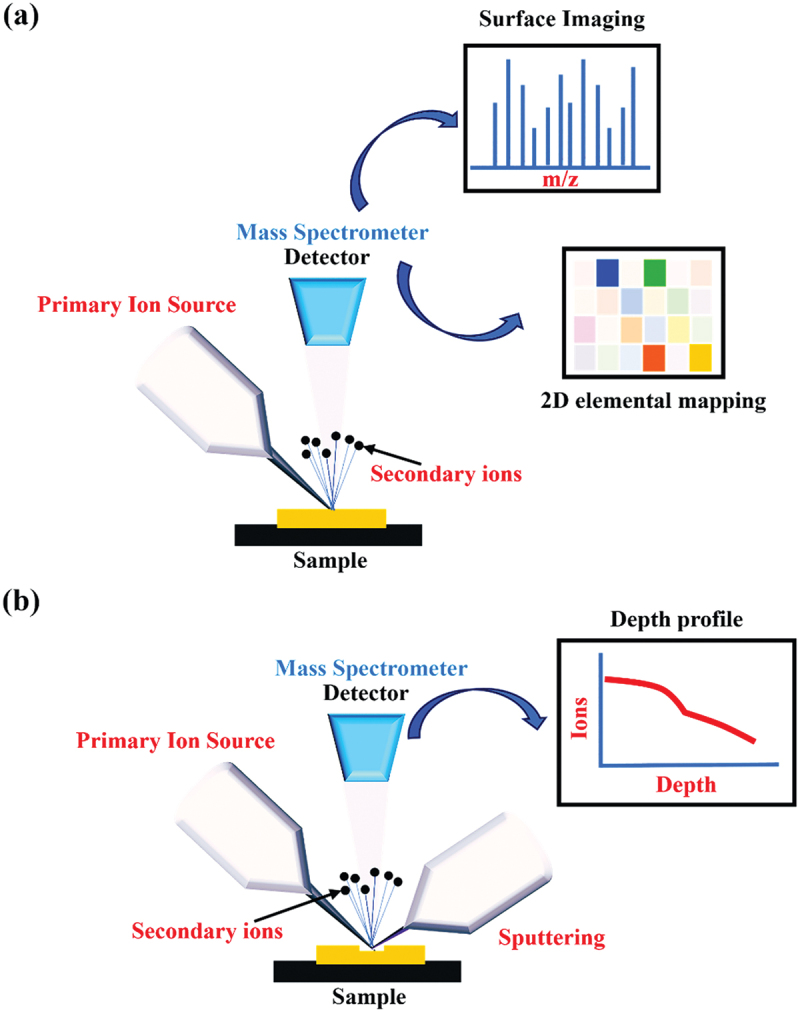
Schematic of (a) surface spectroscopy and surface imaging, followed by (b) depth profiling.

TOF-SIMS operates in three distinct modes (Surface spectroscopy, Surface Imaging, Depth profiling), each catering to specific analytical needs [73–75], as shown in Figure 2. In the Surface Spectroscopy mode, TOF-SIMS provides invaluable elemental and molecular insights into the surface composition of materials [73–75]. This mode boasts an unlimited mass range, ensuring comprehensive detection capabilities, and achieves remarkable sensitivity in the parts per million (ppm) to parts per billion (ppb) range. The exceptional mass resolution, surpassing 10,000, facilitates detailed and precise analysis. While, the surface Imaging, another key mode, enables the parallel detection of masses while offering high lateral resolution, typically less than 100 nm [73–75]. This mode is instrumental in acquiring spatially resolved information about the sample surface, making it indispensable for applications requiring detailed surface characterization. Depth Profiling, the third mode, excels in providing a thorough understanding of the composition of thin layers within a sample. It achieves high depth resolution, typically less than 1 nanometre, and is adept at characterizing layers ranging from 1 nanometre to microns in thickness [73–75]. Utilizing parallel mass detection, Depth Profiling allows for simultaneous analysis of multiple mass fragments during the exploration of a material’s subsurface layers. In the realm of scientific applications, TOF-SIMS serves as a powerful tool for surface and depth analysis. Its capabilities empower researchers to uncover intricate details of elemental and molecular compositions, spatial variations on surfaces, and the layered structure of materials, contributing to advancements across various fields, including materials science, biology, and nanotechnology.

## 3. Depth profile and surface analysis for battery research

It took approximately 30 years from the initial development of SIMS [76] in 1949 until the early 1980s, when SIMS was first employed in battery research. Early application of SIMS to battery research was demonstrated by G.C. Nelson et al. in 1980. Their study on Li/FeS_2_ primary batteries revealed the dominant role of water reaction with Li(Si) alloy during the manufacturing process of thermal batteries [77]. Regarding the analysis of lithium-ion conductors, J.N. Coles et al. first utilized SIMS in 1974 to determine the self-diffusion coefficient of Li^+^ in lithium fluoride [78]. It is important to emphasised here that TOF-SIMS offers distinct advantages over other techniques such as WDX/FIB-SEM, AES, XPS, and sector-type SIMS, making it uniquely powerful for nanoscale chemical imaging and surface-sensitive applications. Its strengths lie in the combination of sub-100 nm lateral resolution (reaching tens of nanometers in advanced systems), high mass resolution (m/Δm > 10,000), and ppm – ppb sensitivity for trace detection, including light elements such as Li. Unlike sequential techniques, TOF-SIMS enables parallel detection of all secondary ions, providing a full mass spectrum at each pixel and allowing retrospective analysis of unknown or multicomponent systems. Depth profiling with low-energy cluster ion beams further enables high-resolution 3D chemical mapping with sub-nanometer depth precision, overcoming artifacts such as beam-induced mixing that often limit sputter-based XPS. Importantly, TOF-SIMS can detect molecular fragments, offering insights into chemical bonding not accessible by AES or WDX and complementing, but extending beyond, the chemical state information of XPS. These capabilities have been demonstrated in mapping Li^+^, SEI components, and degradation products in batteries with nanoscale precision, as well as profiling dopant distributions in semiconductor heterostructures with high spatial and depth resolution, analyses that remain challenging for other techniques [54,79,80]. This section highlights selected case studies on SSBs from SIMS-based battery research accumulated over more than 40 years, illustrating how SIMS has adapted to address the complexities of modern batteries.

### 3.1. Electrodes and electrolytes in lithium-based batteries

In battery research, SIMS has been extensively applied to detect trace elements and study surface and interfacial processes due to its exceptional sensitivity. TOF-SIMS, in particular, has emerged as a vital tool in the development of lithium-based batteries. For anode materials, it has enabled detailed investigation of surface passivation layers, and lithium diffusion behaviour, especially in electrodes made of lithium, aluminium, silicon, and various alloys. TOF-SIMS has also identified features such as lithium distribution gradients, two-phase lithiation processes, and interface modifications that contribute to enhanced performance and stability. While in the case of cathodes, it has revealed important information on lithium-ion transport, material degradation, and the impact of dopants, and surface coatings in systems such as LiFePO_4_, LNMO, and NMC. Moreover, in both solid and liquid electrolytes, TOF-SIMS has proven essential in evaluating lithium transport, interface design, and dendrite suppression. A variety of examples are summarised in Table 1 and further discussed in detail in Anode for anodes, 3.1.2 for cathode and 3.1.3 for electrolytes.

**Table 1.** Application cases of sims measurements in the lithium batteries.

Element in battery	Material	Topic	Ref.
Cathode	Li_1+*x*_(Ni_1−*y*−*z*_Co_*y*_Mn_*z*_)_1−*x*_O_2_ (NMC)	Thermal performance, degradation mechanism, coating and doping effects	[81–112]
	LiFePO_4_	Coating layer evaluation (e.g. PEG additives) and corrosion/impurity analysis	[113–122]
	LiMnO_4_	Lithium insertion and interfacial studies	[111,123–126]
	LiCoO_2_	Structural degradation and ion diffusion	[127–135]
	Li_7_La_3_Zr_2_O_12_ (LLZO)	Garnet-based electrolyte modification and lithium dendrite formation	[136–141]
	Li_4_SeS@C (Li – S)	Cathode uniformity, polysulfide suppression and binder agglomeration impact	[142–144]
	MnO_2_	Lithium insertion mechanisms and stability	[145–151]
	Transition metal-based cathodes such as MoO_*x*_S_*y*_, V_2_O_5_, Fe-based oxides	Transition metal cathode performance	[152–158]
		Degradation mechanisms	[156]
Anode	Lithium metal	LiF/Li_2_CO_3_ passivation and interfacial layer	[49,149,150,159–167]
		Additive role (FEC, electrolyte influence)	[166]
		Bulk reduction on metals	[160]
	Li-Si alloys	Lithium diffusion	[168]
		Elemental Reaction	[77]
	Aluminium-based alloys	Surface passivation; alloy suppression	[78,169–174]
		Adhesion in Al-polymer films	[175]
	Carbon-based (coated/doped)	Surface functionalization	[176–182] (Li_4_Ti_5_O_12_) [162]
	Nickel Aluminium alloys	Lithium concentration gradients	[183]
	Graphite/nitride-based (doped)	Electrochemical stability	[184–189]
	Magnesium-lithium alloys	Deposition mechanism; lithium gradient	[183]
	Nickel/chromium-based alloys	Lithiation – deconversion profile	[184,185]
	Copper/tin-based (e.g. Cu_*x*_O-TiO_2_, Al_2_O_3_-coated Sn)	Ion transport improvement and Fluorinated interface formation	[181,190–193]
	Cobalt-based anodes	Electrochemical depth profiling	[186,194,195]
	Silicon-based (carbon-coated Si, rGO)	Structural stability	[187–189,196,197]
	Germanium nanowires	Lithium segregation, mechanical integrity	[198]
	Bismuth-based alloys	Dendrite suppression	[174,199]
	Iron sulfide anode	Ageing/degradation in LiClO/PC electrolyte	[200]
Electrolyte	La_0.56_Li_0.33_TiO_3_	^6^Li tracer diffusion vs. interfacial insertion influenced by electric fields	[201]
	LiBETI electrolyte with Al current collector	Passive film formation and Li-Al alloying behaviour at the current collector	[173]
	Li_3_PO_4_ thin films	Lithium diffusion coefficients via depth profiling	[202]
	LATP thin films	Compositional uniformity and ionic conductivity changes with annealing	[203]
	LiPON thin films	Effect of structural disorder on lithium-ion mobility	[204]
	Garnet-type solid electrolytes	Moisture-induced degradation and lithium depletion	[205]
	LATP-coated separator	Formation of dense interphases for cathode stability	[206,207]
	MOF with zwitterions	Formation of uniform LiF-rich layer and dendrite suppression	[208]
	LiCl solid electrolyte	Diffusion mechanism linked with electrochemical performance	[209]
	PEO membranes	Lithium diffusion behaviour correlated with ionic transport properties	[210]

#### 3.1.1. Anode

In anodes, TOF-SIMS has established itself as a leading characterization tool due to its unparalleled chemical sensitivity and spatial resolution. By bombarding an anode surface with a pulsed primary ion beam and detecting the resulting secondary ions, TOF-SIMS provides detailed depth profiles and 3D chemical mapping, enabling direct observation of interfacial reactions, lithiation mechanisms, passivation, elemental diffusion, degradation, and protective layer composition. Recent studies demonstrate how TOF-SIMS advances understanding of electrode processes. For instance, Uxa et al. and Erwin Hüger et al. identified a two-phase lithiation mechanism in silicon anodes, showing that Li_0.3_Si dominates diffusion independent of oxygen, with mechanical stress maintaining planar reaction fronts [159,168]. TOF-SIMS further revealed the role of mechanical stress in preserving the planar reaction front and the stability of the Li-poor phase even at full state of charge, while also providing detailed insights into the composition and evolution of passivation films during sputtering. Seok-Gyun Chang et al. observed that passive films form at ~2.0 V vs. Li/Li^+^, and TOF-SIMS uncovered a multilayer structure with a surface layer of alkyl groups, including lithium carbonate, and underlying layers of lithium hydride, lithium hydroxide, and lithium superoxide [160]. A similar study by Svenja-K. Otto et al. confirmed a nanometre-thick bilayered structure [49], and Yuntao Guo et al. used TOF-SIMS to map the gradient distribution of Li solid-phase content across thick electrode cross-sections [161]. Beyond lithiation and passivation, TOF-SIMS has been applied to modified (coated or doped) electrodes: Chung et al. confirmed carbon coatings on Li_4_Ti_5_O_12_ surfaces [162], while Jun Beom Kim et al. showed that Br doping predominantly occurs at particle surfaces, forming conduction pathways that enhance electron hopping and lithium-ion transport, improving anode performance, though excessive Br doping induced secondary phases that reduced performance [163]. Additionally, Austin G. Paul-Orecchio et al. analysed protective layers using TOF-SIMS and XPS, revealing a Li – In alloy combined with a nitrate-derived layer of LiN_*x*_O_*y*_, Li_2_O, and Li_3_N [164], where the mechanically stable and ionically conductive Li – In alloy accounts for the observed enhanced stability and performance.

Similarly, in aluminium, or magnesium-based anodes, SIMS has played a significant role in understanding surface composition and diffusion processes. SIMS depth profiling revealed critical insights into surface enrichment and diffusion. Masanori et al. mapped potassium in β-alumina [169], while Amstutz et al. quantified Mg and Ga distributions in AlMgGa alloys, showing gallium-enriched AlGa films beneath the oxide layer (2–8 at.% vs. 85 ppm) [170]. Textor et al. confirmed ^7^Li^+^ and ^24^Mg^+^ enrichment and determined diffusion coefficients from SIMS profiles [78,171]. TOF-SIMS by Myung et al. showed freshly scratched Al surfaces consist primarily of Al^III^–O compounds, with passive films differing by electrolyte: LiBETI forms thin, less fluorinated layers resisting Li – Al alloying, whereas LiPF_6_ produces heavily fluorinated AlF_3_ layers [172,173]. Jinho Chang et al. studied electrodeposition of Mg – Li, Li – Al, and Ni – Al alloys from molten salts, using TOF-SIMS to verify lithium incorporation in Mg films, higher Li content at negative deposition potentials, and alloy formation during deposition and dissolution, while also probing Mg – Li alloy behaviour in borohydride diglyme electrolytes [183]. Building on TOF-SIMS proven utility, transition metal-based anodes have been extensively characterized, too such as in nickel-based systems, K. Takanashi et al. showed that nickel hydroxide particles are coated with cobalt additives, influencing battery performance [184]. For chromium, Jun-Tao Li et al. revealed via TOF-SIMS that thermally oxidized Cr_2_O_3_ films undergo incomplete conversion, forming an outer Li_2_O-rich layer over an unconverted inner Cr_2_O_3_ region, with volume changes linked to conversion and deconversion reactions [185]. In cobalt-based anodes, TOF-SIMS of electroplated Sn – Co films demonstrated incomplete lithium alloying, producing a lithiated outer layer and a largely non-lithiated inner layer, with alloying-induced cracks fragmenting the uniform layer [186]. Studies on Co – N films indicated that carbon doping suppresses nitrogen out-diffusion, enhancing thermal stability and structural integrity [194,195]. Copper- and tin-based anodes also benefit from SIMS insights: Cu_*x*_O – TiO_2_ nanomaterials showed intermixing with Ti substrates [190], while chlorine-modified TiO_2_ exhibited enhanced lithium insertion and conversion reactions confirmed by TOF-SIMS [191]. TiO_2_(B)/poly(ethylene oxide) solid-state electrolytes formed LiF-rich interphases that facilitated uniform lithium deposition, as demonstrated by TOF-SIMS and Cryo-TEM [192]. Additionally, Ni electrodeposition on nano-twinned Cu foils enhanced oxidation resistance and mechanical strength, with TOF-SIMS revealing surface-localized Ni and reduced oxide layers [193]. Finally, FeS electrodes displayed lithiation-induced grain growth, cracking, and minor capacity fade, with SIMS revealing a duplex structure of organic and inorganic compounds [200].

Extending the earlier discussion on TOF-SIMS investigations of lithium, metal, and transition-metal anodes, there are studies on carbon-based anode materials further demonstrate the technique’s versatility in probing surface chemistry, interfacial reactions, and electrochemical performance across diverse anode systems. In 2006, H. Groult et al. investigated carbon nanoparticles prepared via the electrolysis of molten carbonates [176]. SIMS analysis confirmed the presence of Na and Li on the carbon surface, though quantitative interpretation was challenging due to ionization yield variations influenced by alkaline cations. To address this, intensity ratios of different atoms were analysed, revealing correlations among Li, C, and Na on the surface. Li_2_CO_3_, identified as a lithiated species, facilitated lithium insertion/deinsertion and the formation of passivating surface films, preventing extensive solvent reduction and enhancing cycling efficiency of lithiated graphite electrodes. Heat treatment at 400°C yielded the highest Na and Li concentrations, further elucidating surface composition effects [176]. Similarly, A. Cheriet et al. developed porous SiC layers through electrochemical etching of highly resistive p-type 6 H – SiC, using lithium as a donor and aluminium as an acceptor [177]. SIMS profiles revealed Si and C in a 1.06 ratio, with traces of Al indicating the p-type nature. N. Karar et al. studied Li deposition on multi-wall carbon nanotube (MWCNT)-coated electrodes using TOF-SIMS [178], observing high Li^+^/Li^−^ counts, uniform lithium distribution, and electrolyte degradation post-cycling, accelerated by SnO_2_ coatings. These results highlighted MWCNT electrode’s potential and the need to address long-term stability. Yu-Jin Han et al. functionalized platelet carbon nanofibers (PCNF) and graphitized PCNF (GPCNF) with C_*m*_F_*n*_ groups via C_4_F_8_ plasma, avoiding covalent fluorine intercalation [179]. TOF-SIMS confirmed predominantly semi-ionic C – F bonds, with CF_3_^+^/C^+^ and C_2_F_3_^+^/C^+^ ratios highest in 60 s plasma-treated samples, demonstrating effective surface tailoring. Elahe Yousefi et al. synthesized TiN-C nanocomposites as LIB anodes, revealing via TOF-SIMS nitrogen incorporation into carbon lattices, phase transitions to nitrogen-doped graphene, and impurity removal during heat treatment [211]. Stefania De Rosa et al. investigated HOPG treated with perchloric and sulfuric acids, where TOF-SIMS showed preferential anion intercalation through surface defects, leading to blistering, surface degradation, and unexpected nitrogen-based functionalization [180], emphasizing defect roles in intercalation processes. Erwin Huger explored C/Cu multilayer electrodes, finding through SIMS that lithium partially penetrates copper to reach underlying carbon layers, with trapped Li reducing reversible capacity, while copper protected the structure over 500 cycles [181]. Minjian Gong et al. applied a gradient-lithophilic carbon nanotube framework (CNTF) for lean-lithium metal batteries, achieving uniform lithium deposition and reduced dendrite formation via TOF-SIMS 3D reconstruction, which confirmed a Li_*x*_C_6_ gradient layer that preserved the CNTF’s porous structure and electrochemical accessibility [182].

Building on carbon-based anodes, recent studies have similarly leveraged SIMS to investigate graphite, nitrides, and silicon-based anodes, providing detailed insights into surface chemistry, lithiation behaviour, and strategies to enhance stability and performance. Myung-ho Kong et al. studied plasma-arc-discharge-synthesized polycrystalline silicon particles (80–100 nm) and composites with graphite, using SIMS to detect phosphorus in P-doped silicon, revealing surface and bulk presence of P, PO, PO_2_, and PO_3_ species [187]. Rafael Janski et al. evaluated refractory metal nitrides as lithium diffusion barriers in silicon, showing that 50 nm titanium and tantalum nitride layers effectively impede Li transport, with SIMS providing insights into barrier performance and ion diffusion [188]. Fangmu Qu et al. developed carbon-coated silicon microspheres, where SIMS confirmed uniform coating, mitigating silicon-electrolyte contact, accommodating volume changes, and stabilizing SEI formation, supporting scalable production of high-performance silicon anodes [196]. Advanced TOF-SIMS studies also highlighted the benefits of electroplated metals and reduced graphene oxide (rGO) in improving graphite anodes’ initial Coulombic efficiency (ICE), with Sn/rGO coatings enhancing electron/ion conductivity, stabilizing SEI, and raising ICE to 83.26% compared to 71.10% for unmodified graphite [197]. Yue Feng et al. investigated lithiation in pure and methylated amorphous silicon thin films, showing that methylation introduced nanovoids and gradual lithium gradients, contrasting with sharp biphasic transitions in pure a-Si:H, and TOF-SIMS confirmed frozen lithium profiles, indicating improved structural stability and uniform lithiation [189]. Similarly, in tin and germanium-based anodes, a study by Behdokht Farbod et al. demonstrated that TOF-SIMS analysis highlights the thickness-dependent nature of Li segregation within the Ge nanowires [198]. Collectively, these studies underscore the power of TOF-SIMS to provide depth-resolved insights into surface composition, interfacial reactions, and degradation mechanisms, offering guidance for the rational design and optimization of anodes.

#### 3.1.2. Cathode

This section of the literature review examines SIMS applications in analysing battery cathode materials, emphasizing chemical composition, depth profiling, and performance. Key studies on manganese-based cathodes, such as MnO_2_, revealed lithium insertion mechanisms and the effects of optimal additives on rechargeability. Other transition-metal-based cathodes, including Mo-, V-, Fe-, and Ni-containing materials, were investigated for ion diffusion and structural stability. Lithium iron phosphate (LiFePO_4_) and layered nickel-cobalt-manganese oxides [Li_1+x_(Ni _1−y−z_Co_y_Mn_z_)_1−x_O_2_, NMC] demonstrated improved conductivity, cycling stability, and thermal performance through coatings, doping, and advanced synthesis, as confirmed by surface evaluations. While there are some sulphur-selenium nanocomposites, where TOF-SIMS analysis of Li_4_SeS@C nanocomposite cathodes revealed minimal Se and S species on lithium surfaces, confirming suppression of polysulfide and polyselenide dissolution. Elemental mapping showed uniform Li, Se, and S distribution within carbon nanocages, supporting stable redox reactions and low volumetric expansion (~1.1%) during cycling, enhancing cycle life [142]. Furthermore, SIMS provided insights into interfacial behaviour, ionic migration, and degradation mechanisms, offering valuable guidance for optimizing cathode materials in next-generation energy storage systems.

Starting with manganese dioxide cathode (MnO_2_), it has been extensively studied using SIMS to understand its surface chemistry, lithium insertion, and additive effects. In 1994, P. Fau et al. employed SIMS for qualitative analysis of MnO_2_ films, revealing high surface concentrations of Mn^+^ and MnO^+^ and a homogeneous film growth [145]. Building on this, Manickam Minakshi et al. investigated TiS_2_ (1–5 wt.%) additives in MnO_2_ cathodes, where SIMS depth profiling showed lithium insertion into the bulk rather than conventional protonation, and that excessive additive content dramatically reduced Li^+^ counts, indicating an optimal additive range for electrochemical performance [146]. Further studies on Zn – MnO_2_ cells with KOH electrolyte revealed persistent K^+^ ions even in charged materials [147], while TiB_2_ additives similarly affected lithium distribution with 1 wt.% yielding the highest and 5 wt.% the lowest Li^+^ counts [148]. Investigations using aqueous LiOH confirmed Li^+^ intercalation into MnO_2_ during discharge, with Li_2_CO_3_ surface layers promoting Li^+^ diffusion while inhibiting OH^−^ transport [149]. To address MnO_2_’s proton insertion limitation, Bi_2_O_3_-doped MnO_2_ electrodes were studied, and SIMS depth profiling revealed higher Li^+^ concentrations in the doped material, explaining enhanced discharge capacity and rechargeability through a lithium insertion mechanism [150]. Complementing these studies, Samane Maroufi et al. demonstrated the sustainable fabrication of MnO_*x*_ thin films from spent batteries for pseudocapacitor applications. Thermal isolation and electrodeposition produced 115–137 nm 3D porous films, and TOF-SIMS confirmed mixed Mn^3+^/Mn^4+^ oxidation states, contributing to high specific capacitance (411 F/g) and 92.6% retention over 2500 cycles [151]. Further, Jonathan Op de Beeck and colleagues investigated the nanoscale electrochemical behaviour of LiMn_2_O_4_ (LMO) and MnO_2_ cathodes in all-solid-state micro batteries using a combined approach of Conductive Atomic Force Microscopy (C-AFM) and SIMS [123]. Their work revealed a strong correlation between lithium-rich regions and enhanced conductivity in LMO films, while SIMS mapping also identified phase segregation responsible for local conductivity variations. Extending this approach, they later employed ion-modulated C-AFM with SIMS [124], which showed field-induced lithium migration with accumulation or depletion depending on bias polarity, and emphasized that grain boundaries exhibited enhanced ionic activity due to defect-mediated reduction in migration barriers. Complementary work by Daniel Albrecht et al. [125]. used SIMS to probe the elemental distribution in LMO thin films, where depth profiles revealed uniform lithium, manganese, and oxygen ratios across multilayer structures, confirming stable stoichiometry. Together, these studies underscore the critical role of SIMS in linking local lithium distribution to conductivity, mapping nanoscale heterogeneities, and providing structural insights that guide the optimization of Mn-based cathode quality and performance in lithium batteries.

While Mn-based cathodes have provided valuable insights into ion distribution and surface chemistry, extending TOF-SIMS analysis to other transition-metal cathode systems further broadens our understanding of elemental migration and interfacial stability across diverse chemistries. For molybdenum systems, Golodnitsky et al. (2005) first used TOF-SIMS to reveal the amorphous nature and depth-dependent stoichiometry of electrodeposited MoO_*y*_S_*z*_ thin films, detecting mixed O – S poly-ion clusters and interfacial Ni silicide formation that improved adhesion [152]. Yufit et al. (2007) expanded this work, with TOF-SIMS confirming the homogeneous submicron distribution of MoO_*y*_S_*z*_ fragments and highlighting oxygen depletion with depth and consistent S/O ratios [153]. Later, Paste et al. used TOF-SIMS to probe LiTFSI dissociation in polymer electrolytes with MoO_3−*x*_ nanobelts, while Huang et al. employed it to identify dominant H^+^ intercalation in oxygen-deficient α-MoO_3_, linking interfacial chemistry to ultrafast energy storage [212,213]. For vanadium oxides, TOF-SIMS depth profiling by Alamarguy et al. demonstrated lithium retention near V_2_O_5_/substrate interfaces after cycling, with subsequent work by Swiatowska-Mrowiecka et al. showing how aging traps Li in duplex V_2_O_5_/V oxides and broadens its distribution with cycling [154,155]. Anand et al. further applied TOF-SIMS surface imaging to reveal degradation pathways at low C-rates in solution-processed V_2_O_5_ [156]. In iron oxides, Tian et al. used TOF-SIMS to spatially resolve lithium diffusion rates across different electrode regions, distinguishing converted (Li_2_O + Fe) from unconverted (Fe_2_O_3_) matrices and revealing slower transport in the former [157]. While in Fe – Cr oxides, TOF-SIMS identified reduced lithium retention in the bulk, clarifying the limited activity of chromium [158]. For LiFePO_4_, TOF-SIMS depth profiling confirmed homogeneous Al-doping [113], detected nonuniform polymer coatings [114,115], and revealed how PEG additives enhance Li solubility within PPy/PEG – LiFePO_4_ composites [115]. It also clarified surface bonding modifications by N and S [116], impurity-phase – driven corrosion [117], interdiffusion across Ti vs. TiN interlayers [118,119], and uniform Li insertion at high C-rates [120], while supporting studies on lithium recovery from spent batteries [121]. Similarly, in LiCoO_2_, TOF-SIMS validated the protective effects of ZrO_2_ and Al_2_O_3_ coatings against electrolyte-induced degradation [127,128], mapped elemental distributions in Mg-doped, titanate-coated films [129], and quantified lithium diffusibility across LWO, LTaO, and LNbO modifications, linking fast diffusion to reduced interfacial resistance [130,131]. Beyond conventional cathode roles, TOF-SIMS imaging also illuminated resistive switching in Li_*x*_CoO_2_, directly capturing lithium migration and reintercalation during SET/RESET operations [132], while tracer diffusion studies (step-isotope exchange) established reliable lithium diffusivity values in c-axis – oriented LiCoO_2_ [133]. More recently, TOF-SIMS depth profiling confirmed interfacial stabilization via NbO_*x*_ coatings [134], and SIMS provided nanoscale lithium/cobalt mapping in ALD-grown Li_*x*_Co_*y*_O_*z*_ thin films [135].

Beyond general transition-metal compositions, TOF-SIMS investigations on layered oxide cathodes, including LiNi_0.5_Mn_1.5_O_4_ and Li_1+x_(Ni _1−y−z_Co_y_Mn_z_)_1−x_O_2_, offer a more detailed picture of structural anisotropy, revealing how diffusion pathways and degradation phenomena evolve in architectures that dominate commercial applications. The high-voltage LiNi_0.5_Mn_1.5_O_4_ (LNMO) spinel is considered a promising LIB cathode; however, LNMO/graphite full cells suffer severe capacity fading due to Mn dissolution. Nicholas P. W. Pieczonka et al. systematically examined Mn and Ni dissolution under varying states of charge (SOC), temperature, storage duration, and LNMO crystal structure, revealing through TOF-SIMS that dissolution stabilized after 40 days at 60°C and decreased significantly at lower SOC, with a tenfold reduction in Mn dissolution and negligible Ni dissolution at 0% SOC compared to 100% SOC [38]. TOF-SIMS further identified metal fluorides (LiF, MnF_2_, NiF_2_) and organic fragments (C_2_H-, CH-, and PO^3-^), evidencing surface decomposition products that elevate impedance but may also form protective interphases against HF attack [81]. Building on these findings, Michael Gellert et al. used TOF-SIMS to analyse LNMO films coated with solid electrolytes, demonstrating diffusion of transition metals into Li_4_Ti_5_O_12_ but stable interfaces with LiNbO_3_, which showed superior stability and lower resistance, while ZrO_2_ exhibited poor separation and higher resistance [82]. Complementary work by Sebastian Kraas et al. employed TOF-SIMS depth profiling in negative ion mode, detecting a ~50 nm surface layer enriched with species suppressing MnO^−^ signals on cycled electrodes [83], whereas Jong Heon Kim et al. revealed interdiffusion between stainless steel substrates and LNMO thin films annealed at ≥ 600°C, highlighting processing-dependent interfacial reactions [84]. Moreover, Ortal Tiurin et al. demonstrated, via TOF-SIMS, deep lithium diffusion into ALD-coated LNMO using Hfac-derived LiF – CF_*x*_ hybrid layers, effectively reducing Mn/Ni dissolution and enhancing stability [85]. Daniel Uxa et al. combined SIMS with ^6^Li tracer studies to quantify lithium self-diffusion in bulk LNMO, finding Arrhenius-type diffusivities with an activation enthalpy of 0.97 ± 0.05 eV, corresponding to lithium vacancy migration [86]. Extending interfacial insights, Lars Pateras Pescara et al. identified, through TOF-SIMS, a fluoride-rich AlF_3_ passivation layer at the LNMO – Al current collector interface, strongly correlated with impedance growth, which was mitigated by LiNbO_3_ coatings [87]. Likewise, Min Xu et al. applied a dual LaNiO_3_/Li_3_PO_4_ coating, with TOF-SIMS confirming uniform Li_3_PO_4_ passivation and coherent LaNiO_3_ integration into the LNMO lattice, collectively reducing fluorine-rich degradation products, suppressing phase transitions, and improving charge transfer kinetics [88].

Whie in case of NMC cathodes, they have attracted extensive attention due to their high energy density, thermal stability, and tunable compositions for specific applications. Yabuuchi et al. (2011) investigated Li_*x*_Co_0.13_Ni_0.13_Mn_0.54_O_2-δ_ synthesized via electrochemical oxidation/reduction, using SIMS to map chemical species on the electrode surface, revealing that lithium was not exclusively sourced from oxide particles and that lithium-containing deposits partially covered the oxides, highlighting slow nucleation of oxygen reduction products like Li_2_CO_3_ at the micrometre scale [89]. Building on this, Hong et al. applied boron and aluminium coatings on LiNi_1/3_Co_1/3_Mn_1/3_O_2_ powders and, via SIMS, confirmed uniform [B, Al]_2_O_3_ layers that suppressed cathode – electrolyte reactions and enhanced electrochemical performance [90]. Katharine R. et al. employed TOF-SIMS depth profiling to study doped Ni_0.5*x*_M_*x*_O_*4*_ spinels, finding surface lithium deficiencies and manganese enrichment, with nickel or dopant ions most enriched, emphasizing the sensitivity of TOF-SIMS in surface compositional analysis [91]. Similarly, Jo et al. demonstrated Li_3_PO_4_ coatings on LiNi_0.6_Co_0.2_Mn_0.2_O_2_ (NMC622) electrodes, where TOF-SIMS detected LiP^+^, LiPO^+^, and Li_2_PO_2_^+^ fragments and showed scavenging of HF and water, reduction of byproducts, improved capacity retention, rate capability, and preserved structural integrity [92]. Subsequent TOF-SIMS studies on silica- and silicon-phosphate-coated Li_0.3_Ni_0.7_Mn_0.3_O_2_ revealed stable coatings and delayed phase transformation under heating (250–600°C), demonstrating suppression of oxygen evolution and surface degradation [93]. Heidy Visbal et al. highlighted DLC coatings on LiNi_0.8_Co_0.15_Al_0.05_O_2_, where TOF-SIMS showed reduced formation of sulphur and phosphorus oxides, lowering interfacial resistance and enhancing cycle life [94]. Neudeck et al. analyzed organophosphate-modified NMC622 surfaces, with TOF-SIMS confirming phosphate ester fragments, revealing that TMSP produced thicker uniform coatings while TNPP provided better chemical stability and cycling performance [95]. Bessette et al. utilized TOF-SIMS to quantify lithium distribution in LiNi_0.5_Co_0.3_Mn_0.2_O_2_ (NMC532) cathodes, addressing matrix and edge effects, detecting hotspots, and linking morphological changes to capacity fade [96]. Dannehl et al. and Hoskins et al. used SIMS and TOF-SIMS to evaluate Al_2_O_3_ coatings on Li-rich NMC and LiNiCoMnO_2_ (NMC111), showing variations in coating uniformity, revealing selective deposition, and correlating coating morphology with electrochemical improvements [97,98]. Kim et al. optimized CNT incorporation in Ni-rich NMC622, with SIMS confirming uniform lithium distribution and enhanced electrode performance [99]. TOF-SIMS studies on lithium borate-coated LiNi_0.8_Co_0.1_Mn_0.1_O_2_ (NMC811) and Li_2_CO_3_/LiNbO_3_ hybrid-coated NMC622 confirmed uniform coatings, reduced cation mixing, suppressed degradation, and improved stability in SSBs [100,101]. Zhang et al. revealed that water-treated single-crystal NMC formed Li_2_CO_3_-rich CEI layers, mitigating transition-metal dissolution as verified by TOF-SIMS, enhancing cycling stability and rate capability [102]. Uxa et al. used SIMS to investigate lithium tracer diffusion in LiNi_0.3_Co_0.3_Mn_0.3_O_2_ (NMC333), showing vacancy-driven diffusion with activation energy of 0.85 eV, and highlighted discrepancies with electrochemical measurements [103]. TOF-SIMS also elucidated boron doping effects in Ni-rich NMC811, revealing surface enrichment that stabilizes the structure and prevents rock-salt formation [104], while revealing SOC heterogeneity in NMC532 due to non-uniform delithiation and microcracking [105]. Zhang et al. applied oCVD PEDOT coatings on NMC811, with TOF-SIMS confirming prevention of cathode – electrolyte interphase formation, significantly improving cycling retention [106]. Kochetkov et al. investigated interfacial stability of lithium-metal chloride solid electrolytes with Ni-rich NMC using TOF-SIMS, identifying severe degradation in Li_2.5_Y_0.5_Zr_0.5_Cl_6_ and superior stability in Li_2_In_1/3_Sc_1/3_Cl_4_ [107]. Hüger et al. resolved discrepancies in lithium diffusivities in NMC111 by SIMS depth profiling, detecting lateral variations, two-phase delithiation, and linking tracer and chemical diffusivities through thermodynamic factors [108]. Finally, lithium tracer studies in nanocrystalline and amorphous NMC films revealed that crystalline order facilitates vacancy-mediated diffusion with lower activation energy, whereas amorphous films hinder transport, with TOF-SIMS depth profiling distinguishing these mechanisms and lateral heterogeneities [109]. Collectively, these studies highlight TOF-SIMS as a critical tool for elucidating surface chemistry, lithium distribution, coating effectiveness, phase evolution, and diffusion mechanisms in transition metal based and NMC cathodes, enabling informed strategies to enhance electrochemical performance and structural stability across diverse material systems.

#### 3.1.3. Electrolyte

For oxide- and garnet-based electrolytes, depth profiling of La_0.56_Li_0.33_TiO_3_ revealed that lithium insertion at interfaces is significantly faster than bulk diffusion, driven primarily by local electric fields [201]. Similarly, studies on lithium aluminium titanium phosphate (LATP) thin films demonstrated uniform composition across the film thickness, with elevated lithium concentrations near the surface due to migration during deposition and annealing, as shown by TOF-SIMS analysis [203]. Zhao Yan et al. further illustrated that LATP-coated separators form dense interphase layers that inhibit transition metal migration, enhancing cycling stability and discharge capacity retention [206]. Moreover, Garnet-type electrolytes such as Li_6.55_Ga_0.15_La_3_Zr_2_O_12_ (Ga-LLZO) and Ta-/Al-substituted Li_7_La_3_Zr_3_O_12_ (LLZO) have also been investigated with TOF-SIMS. Proton-lithium exchange in Ga-LLZO was shown to create a lithium-depleted layer extending over 1 μm, highlighting surface degradation effects on bulk lithium transport [136,205]. While Sr doping of LLZO suppressed lithium segregation at grain boundaries and created homogeneous conduction pathways, as spatially resolved by TOF-SIMS, corroborating improvements in ionic conductivity [137]. Subsequently, Ta- and Al-substituted LLZO films deposited on stainless steel were characterized via SIMS, showing increased lithium incorporation at higher substrate temperatures. This uniform Li distribution improved cathode – electrolyte contact and ionic transport, highlighting the role of film composition and deposition parameters in optimizing solid-state cathode performance [138]. Also, SIMS-based studies of lithium dendrite formation in LLZO and ultrafast-sintered LLZO revealed dendritic growth along intergranular regions and surface Li_2_O accumulation, emphasizing the importance of defect control and post-sintering treatments for stable solid-state electrolyte performance [139,140]. Complementary investigations on LiPON thin films further confirmed the influence of structural disorder and interface engineering on lithium-ion mobility [204].

In polymeric electrolytes, TOF-SIMS provides high-resolution insights into lithium transport and interfacial phenomena that influence battery efficiency and safety. Studies on sulfurized poly(acrylonitrile) (SPAN)-based cathodes demonstrated that elevated drying temperatures cause PVdF binder migration, increasing charge transfer resistance, while poly(acrylonitrile)-based systems maintained homogeneous distributions and stable performance [143]. β-Li_3_PS_4_ solid electrolytes combined with polymeric binders showed that hot pressing produced a thicker (~6 μm) binder-rich layer, enhancing mechanical stability and improving lithium-ion transport through uniform PS_4_^3-^ distribution [144]. Further modifications using zwitterion-functionalized MOFs facilitated uniform LiF-rich SEI formation, suppressing dendrites and stabilizing lithium-ion transport [208]. Strategies incorporating electron-withdrawing groups in composite polymer electrolytes selectively anchored TFSI^−^ ions, yielding Li^+^ transference numbers as high as 0.91, highlighting the synergy between chemical functionalization and interfacial engineering as captured by TOF-SIMS [165]. Similarly, Angelina Jocic et al. applied it to photoluminescent porous polymers, verifying chemical purity by detecting the absence of catalyst residues, confirming complete polymerization of brominated monomers, and mapping uniform distribution of functional groups [214].

TOF-SIMS also plays a central role in understanding interfacial chemistry in liquid electrolytes and their impact on lithium-metal electrodes. Investigations on LiBETI electrolytes revealed that aluminium develops a protective passivation film, mitigating corrosion and enhancing stability when paired with graphite or Nano-SnO_2_ active materials [173]. Fluoroethylene carbonate (FEC) decomposition was shown to generate LiF on lithium surfaces, stabilizing Li electrodes in Li-S cells [166]. Functional additives such as pentafluoro styrene promoted the formation of gradient interfacial layers rich in C-F and LiF/Li_3_N, facilitating uniform lithium deposition and extended cycling stability over 3000 hours in symmetric Li/Li cells [167]. Additionally, the mixed crystal solid electrolyte 15NaI·LiBH_4_ was demonstrated to exhibit predominantly Li^+^ conduction despite minor Na^+^ content, with TOF-SIMS depth profiling confirming lithium as the dominant charge carrier and emphasizing the critical role of interface engineering in Na-based materials [215]. These findings collectively underscore TOF-SIMS as a versatile analytical tool capable of resolving chemical and spatial distributions across solid, polymeric, and liquid electrolyte systems, directly informing strategies to enhance lithium-ion transport, interfacial stability, and battery lifespan.

### 3.2. Overcharge tolerance, stability, and recycling

TOF-SIMS has emerged as a crucial tool for elucidating complex mechanisms across lithium-based battery systems, too. It provides powerful insights into overcharge tolerance, thermal stability, recycling efficiency, and corrosion resistance. In polymer lithium-ion cells, TOF-SIMS revealed that overcharging results in non-dendritic lithium deposition on carbon anodes, forming weak electronic conduction paths that redirect part of the overcharge current, without damaging the cell. A sharp lithium peaks near the surface confirmed this localized deposition, and post-air exposure analysis distinguished intercalated lithium from reactive lithium products, shedding light on environmental interactions [216]. Addressing thermal and durability challenges, TOF-SIMS confirmed the presence of MgO and Al_2_O_3_ coatings on LiCoO_2_ cathodes, enhancing thermal stability [217], while in Li-S batteries, it revealed the formation of partially lithiated LiFeS_2_ and suppressed polysulfide shuttle effects by showing uniform elemental distributions [218]. TOF-SIMS also linked lithium plating and SEI degradation to thermal gradients, showing lithium/phosphorus migration into the SEI at higher temperatures [219]. In battery recycling, TOF-SIMS mapped lithium distribution in pyrometallurgical slags, identifying lithium incorporation into LiAl and spinel phases, with optimal recovery seen via carbonate and oxide formation [220,221]. In LMBs, LiF-rich interphases within separators were visualized, which suppressed dendrite formation and improved safety [222]. It also characterized CEI layers in metal-free organic batteries, where additives like tris(trimethylsilyl) borate enhanced stability and minimized degradation [223]. For corrosion and passivation studies, TOF-SIMS identified (Cr, Fe)-fluorides as superior passive layers on stainless steel in LiPF_6_ electrolytes [224], and showed AlF_3_ and TiF_4_ formation on aluminium and titanium, offering high electrochemical stability [225]. Carbon coatings were found effective in shielding LiFePO_4_ against HF-induced corrosion [226], while in TiO₂ anodes, TOF-SIMS detected titanium oxy-fluorides, with HF scavengers improving cycling durability [227]. Collectively, these studies demonstrate TOF-SIMS’s indispensable role in battery research by providing precise chemical, spatial, and depth-resolved data critical for optimizing battery design, performance, safety, and sustainability.

### 3.3. Role of TOF-SIMS in sodium, potassium, zinc, air- and anode batteries

TOF-SIMS has also become a key technique in various other type of batteries such as sodium battery, offering detailed insights into anodes, cathodes, electrolytes, and SEI layers, too. Starting with anodes in sodium batteries such as tungsten trioxide (WO_3_). TOF-SIMS characterization of WO_3_ by Francisco Jose García-García et al. revealed efficient sodium ion penetration into the film’s porous nanocolumnar structure, driving a transition from amorphous to crystalline phases, thereby highlighting the importance of structural optimization for stable, high-performance sodium storage [228]. Complementing this, Behdokht Farbod et al. reported on various ternary Sn-Ge-Sb thin film alloys for NIBs, where TOF-SIMS provided insights into the sodiation-induced volume expansion of up to 300–400% and revealed that while sodium segregation occurs, heavily alloyed systems show reduced interfacial softening, suggesting that substitutional solid solution strengthening and multiphase nanocomposite microstructures enhance resistance to delamination and improve cycling stability [229]. In parallel, R. Väli et al. demonstrated that D-glucose-derived hard carbon (GDHC) anodes for LIBs and NIBs display distinct SEI compositions, with TOF-SIMS revealing a more inorganic SEI in sodium systems and pronounced elemental distribution changes upon cycling, highlighting the effect of SEI chemistry on performance [230]. TOF-SIMS further elucidates the role of defect-rich lignin-derived skeletal carbon nanofibers in promoting NaF-rich SEI formation, uniform ion transport, and dendrite suppression [231], and similarly, Na segregation at the Si/graphene interface correlates with enhanced Na storage and cyclic stability in Si-based anodes [232]. On the cathode side, Liang Deng et al. confirmed via TOF-SIMS the formation of a dense, uniform interfacial layer for Na_3_V_2_(PO_4_)_2_F_3_, improving charge-transfer kinetics and temperature adaptability [233], while Li/F co-doping in O_3_-type NaNi_0.45_Mn_0.4_Ti_0.1_Co_0.05_O_2_-0.08LiF (P2-LNMTCOF) cathodes revealed, through TOF-SIMS, strengthened TM-F bonds stabilizing TM-O(F) octahedra and mitigating voltage drops [234]. Xiangyu Ding et al. demonstrated that cubic NaMn hexacyanoferrate (NaMHCF) with low water content exhibits uniform Na and Fe(CN)_6_ distribution and minimal side reactions, as confirmed by TOF-SIMS depth profiling and 3D mapping [235], while Ru substitution in P2-type Na_0.67_Mn_1-X_Ru_X_O_2_ cathodes enhances Na^+^ distribution, surface stability, and cycling performance, with TOF-SIMS identifying the optimal doping level to prevent Na_2_RuO_3_ formation [236]. For Na_3_V_2_(PO_4_)_3_/C (NVP/C) electrodes, TOF-SIMS highlighted chemical degradation on anodes and stable SEI layers on cathodes, correlating with performance differences [237], and in NVPF cathodes, TOF-SIMS confirmed the formation of a multilayered CEI that enhances long-term cycling in H-NaODFB electrolytes [238]. Regarding electrolytes, TOF-SIMS analysis revealed selective Na^+^ deposition at glass/platinum interfaces [239], bilayer Al passivation on current collectors [240], and protective Na_*x*_BO_*y*_F_*z*_ CEI formation in hybrid dual-salt polymer electrolytes [241], underscoring the critical role of interphase chemistry. In solid-state systems, Ziming Ding et al. showed that Na filament growth in Na-β″-alumina is grain-boundary dependent, with TOF-SIMS mapping revealing segregation along voids and triple junctions that drive mechanical and ionic instability [242]. For SEI studies, TOF-SIMS revealed uniform, multilayered SEI structures on sodium metal formed with NaBH_4_/ether-based electrolytes, dominated by NaH and NaBO_2_, enhancing dendrite suppression and cycling life [243–245]; similar analyses on hard carbon, Sb, phosphorus, and red phosphorus electrodes highlighted SEI composition and evolution as functions of electrolyte chemistry, additive use, and cycling conditions [246–249]. TOF-SIMS further demonstrated that FEC-containing electrolytes and composite gel polymer electrolytes create NaF-rich, uniform, and thinner SEI layers, promoting interfacial stability, mitigating dendrite growth, and enhancing electrochemical performance [250–252]. These findings, as summarized in Table 2, underscore the pivotal role of TOF-SIMS in advancing sodium battery systems by enabling precise characterization of interfacial chemistry, ion transport, and material stability.

**Table 2.** Application cases of sims measurements in the sodium batteries.

Element in battery	Material	Topic	Ref.
Cathode	Na_3_V_2_(PO_4_)_2_F_3_	Interfacial layer formation	[233]
	NaNi_0.45_Mn_0.4_Ti_0.1_Co_0.05_O_2_-0.08 LiF	Granular insights into binding states and structural transformations	[234]
	Manganese hexacyanoferrate	Structural stability	[235]
	Ruthenium-doped cathodes	Map sodium concentration and defect density	[236]
	Na_3_V_2_(PO_4_)_3_/C	Surface heterogeneity and chemical degradation mechanisms	[237,238]
Anode	WO_3_	Sodium ion penetration	[228]
	Ternary Sn-Ge-Sb thin film alloys	alloy design’s critical role in improving resistance to crystallization and interfacial delamination	[229]
	D-glucose-derived hard carbon and defect-rich lignin-derived skeletal carbon nanofibers	SEI and dendrite formation	[230,231]
	Silicon/graphene interfaces	SEI	[232]
	Bilayer passivation film on aluminium	Passivation layers	[240]
	Sb films and phosphorus	Phase distributions, sodiation-induced stresses, and SEI composition	[247–249]
	spray-coated hard carbon	degradation	[250,252]
Electrolyte and its interfaces	Mixed alkali borosilicate glasses	Selective sodium transport	[239]
	Dual-salt polymer electrolytes	CEI	[241]
	NaBH_4_-based electrolytes	Multilayered SEI dominated by NaH and NaBO_2_	[243,245]
	Glyme-based electrolyte	Decomposition of solvent molecules	[244]
	Carbonate-based systems	SEI	[246]
	Fluoride-rich SEI layers	Degradation	[250–252]

Furthermore, TOF-SIMS has increasingly contributed to the study of potassium-, zinc-, and air-based battery systems, where R. Mills et al. first applied TOF-SIMS to identify KH, KHCO_3_, and K in K_2_CO_3_ aqueous electrolysis, revealing hydride clusters and confirming KH stability at 600°C, with positive and negative ion spectra differentiating K- and H-containing species, thereby providing fundamental insights into inorganic hydride formation and stability [253]. Extending its utility, TOF-SIMS was employed by Reona Miyazaki et al. to trace lithium deposition in the KI-KBH_4_-LiI solid solvent system, confirming lithium-ion transport via interstitial mechanisms and mapping the contributions of Li^+^, K^+^, and I^−^ to conduction, highlighting how dense microstructure and high plasticity reduce grain boundary resistance and stabilize ionic transport [254]. Similarly, Miao Xie et al. utilized TOF-SIMS depth profiling to demonstrate that K(H_2_O)MoS_2_ preserves layered structures, stabilizes Mo-S bonds, and mitigates electrolyte decomposition through intercalated hydrates, with depth profiles showing consistent MoS_2_ fragments and MoSO/SH^−^ species [255]. In zinc-ion batteries, TOF-SIMS elucidated hybrid ZnSO_4_–NaCl electrolyte effects, showing suppression of dendrites and formation of NaZn_4_(SO_4_)Cl(OH)_6_·6 H_2_O rather than Zn_4_SO_4_(OH)_6_·4 H_2_O [256], and revealed DMSO-induced SEI enrichment with S^−^ and OH^−^ species that promoted (101)-oriented Zn plating [257]. Further, Xiaoyun Xu et al. demonstrated that a bionic ion-pump interface comprising acetylated proteins forms uniform organic (CONH^−^) and inorganic (ZnF_2_, ZnS) layers, facilitating Zn^2+^ transport and dendrite suppression, as confirmed by TOF-SIMS depth profiling [258]. In anode-free and air batteries, TOF-SIMS analysis by Svetlana Menkin et al. highlighted SEI composition on copper substrates, showing LiF, copper oxides, and LiPF_6_-derived species govern preferential Li plating [259], while studies on lithium-air systems by Akiya Karen et al. distinguished reaction products such as Li_2_O_2_ and Li_2_CO_3_ on discharged cathodes [52]. Similarly, TOF-SIMS mapping in manganese nitride electrodes revealed O/N gradients that inform Zn-air electrode design [260], and analysis of Na_2_S additives in iron-based air batteries showed sulphur-enriched oxide layers enhance stability and suppress hydrogen evolution [261]. Finally, TOF-SIMS combined with isotope-labelling in Li-O_2_ systems elucidated the Li_2_O_2_/electrolyte interface as the primary oxygen evolution site [262], enabled monitoring of Br^−^/Br_3_^−^ redox mediator effects on Li_2_O_2_ decomposition [263], and revealed uniform Li_2_CO_3_ byproduct distribution within discharge deposits, offering critical insights to mitigate high-voltage overpotentials [264]. These findings are summarized in Table 3.

**Table 3.** Application cases of sims measurements in the potassium, zinc, air- and anode batteries.

Battery type	Material	Topic	Ref.
Potassium-ion batteries	K_2_CO_3_ aqueous electrolyte	Identification of KH, KHCO₃ and hydride compound stability at high temperature	[253]
	KI-KBH_4_-LiI	Ion conduction, interstitial migration	[254]
	K (H_2_O) MoS_2_	Interfacial species	[255]
Zinc-ion battery	ZnSO_4_-NaCl hybrid electrolyte	Suppression of dendrites	[256]
	DMSO additive	SEI	[257]
	Zn@BIPI/a-HPace interface	Stable SEI formation	[258]
Anode-free lithium battery	Cu current collector	SEI composition and Li plating morphology	[259]
Lithium-air battery	Carbon electrode	Reaction product identification (Li_2_O_2_, Li_2_CO_3_) and isotope labelling	[52]
Zinc-air battery	Mn_3_N_2_ electrode	depth profiling and composition gradient	[260]
Iron-air battery	Na_2_S additive	Suppression of hydrogen evolution; porous sulphur-enriched layer via TOF-SIMS	[261]
Lithium-oxygen battery	Li_2_O_2_/electrolyte interface	Isotope labelling	[262]
	Discharge products with Br^−^/Br_3_^−^ mediator	Uniform Li_2_CO_3_ distribution	[263,264]

### 3.4. Ex-situ SIMS analysis of interphase formation and degradation mechanisms

**Figure 3.**
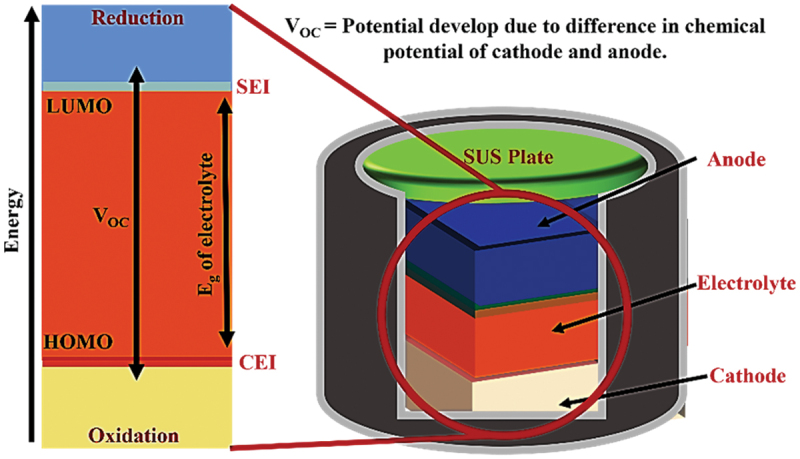
Schematic of the SEI and CEI formation mechanism.

In particular, the solid – electrolyte interphase (SEI) and cathode – electrolyte interphase (CEI) are recognized as key layers that critically influence overall battery performance. The formation of SEI and CEI layers occurs due to the reduction and oxidation of electrolyte components on electrode surfaces. When the lowest unoccupied molecular orbital (LUMO) of an electrolyte component is lower than the anode’s Fermi level, insoluble reduction products deposit to form the SEI layer[4,26,252,265], as shown in Figure 3. Similarly, components oxidize when their highest occupied molecular orbital (HOMO) exceeds the cathode Fermi energy, resulting in a CEI layer. SEI layers exhibit heterogeneous and complex substructures, with several structural models proposed over the decades [266–269]. Among them, the ‘multilayer model’ proposed by Zaban et al. [270] is currently the most widely accepted. Siqi Shi et al. investigated this model by combining isotope diffusion techniques with SIMS. Based on their results, they proposed a ‘two-layer/two-mechanism model’, in which the SEI consists of a porous organic outer layer and a compact inorganic inner layer, each exhibiting a different mechanism for lithium-ion transport [271]. In multilayered SEI models, Li^+^ diffusion follows distinct mechanisms such as interstitial migration in the compact inner layer and co-diffusion with anions in the porous outer layer, varying with voltage conditions [271–274]. It is important to mention here that generally, the SEI layers (30–100 Å) are thicker than cathode electrolyte interphases (CEIs, 5–10 Å), reflecting more extensive reactions at the anode [275,276].

In interphases, TOF-SIMS has been pivotal in directly visualizing SEI/CEI structures and dynamics since the early 1900s [266,277], revealing that Li^+^ transport follows Fickian diffusion in the organic-rich outer SEI and a knock-off mechanism in the crystalline inner layer [271]. It further identifies LiF, Li_2_CO_3_, and Li_2_O as dominant SEI components [268,278–283], with Siqi Shi et al. confirming the two-layer/two-mechanism diffusion model [271]. Chengwei Ma et al. demonstrated that SEIs evolve into denser, inorganic-rich forms that enhance passivation on Li metal [284], while coatings such as Li_2_CO_3_ and Li_3_N stabilize interfaces [285], and LiF-rich SEIs formed in THF electrolytes improve retention [286]. TOF-SIMS studies also extend to electrolytes, where systems such as LiClO_4_ [272], LiCoPO_4_ [287], Li_4_Ti_5_O_12_ [288–290], Lithium bis-(trifluoromethanesulfonyl)imide (LiTFSI) [291,292], Li_2.96_P_0.98_S_3.92_O_0.06_-Li_3_N glass-ceramic electrolyte [293], and Li_6_PS_5_Cl solid electrolytes [294–296] have been used to probe SEI evolution, often showing improved stability with additives [297,298] like LiF_2_BC_2_O_4_ (LiDFOB) additive [299], lithium difluoro(bisoxalato) phosphate (LiDFBOP) [300–302], Trimethylene Sulfite [303,304], 2,3-dimethylmaleic anhydride (DMMA) [305], triphenylphosphine selenide [306], and C_3_H_3_FO_3_ (FEC) [307,308]. Additives such as TNNT and tert-pentanol further enhance ionic conductivity [309], while Li_3_PO_4_ interlayers suppress side reactions in solid-state systems [310]. TOF-SIMS also uncovers the role of fluorinated compounds in promoting uniform, LiF-rich SEIs [311–314], as well as the beneficial effects of MXenes [315,316], hybrid interfacial layers [317–319], and advanced polymers such as redox-active, photoluminescent porous FTN-based systems [214,320]. Beyond interphase chemistry, TOF-SIMS profiling has elucidated SEI integrity in graphite [311,321–326], silicon-based anodes [55,327–343], and transition metal-based systems [157,185,186,190,200,344–352], where strategies like carbon/tin coatings and prelithiation improve performance [55,337–343]. In Ni-, Mn-, and Fe-based electrodes, it reveals transition-metal crossover catalyzing SEI degradation and lithium inhomogeneity, as shown by Sim et al. [350], while high-voltage electrolytes mitigate these issues by forming thinner, more stable SEIs and reducing Ni/Mn deposition. Collectively, TOF-SIMS insights into SEI/CEI composition, evolution, and interfacial phenomena provide critical guidance for designing next-generation batteries with improved stability, conductivity, and safety.

In liquid and polymer-based electrodes and electrolytes, TOF-SIMS plays a crucial role in probing SEI and CEI formation, and evolution, providing insights into ion transport pathways, interfacial stability, and additive effects. Peng Lu and Stephen J. Harris investigated a LiClO_4_-derived SEI film on copper using TOF-SIMS, showing that the SEI contained an ~5 nm porous interfacial region enabling electrolyte diffusion, beneath which a compact Li_2_O/Li_2_CO_3_ layer restricted electrolyte penetration while Li^+^ transport occurred via ion exchange [272]. Complementary work by V. Winkler et al. employed TOF-SIMS sputter depth profiling to analyse SnO_2_ nanoparticle anodes cycled in LiTFSI-based electrolytes with VC and FEC additives, revealing that carbonate SEI components accumulated mainly within the top 10–15 nm, with cracks exposing reactive sites where further SEI growth occurred; upon aging, homogeneous lateral distributions of Li^−^ and carbonate species extended deeper, correlating with continuous electrolyte decomposition and reduced Coulombic efficiency [291]. Similarly, Thomas Meyer et al. used isotopic ^6^Li/^7^Li labelling with TOF-SIMS to probe polymer – ceramic composite solid electrolytes, showing preferential lithium migration through LLZTO ceramic fillers and strong accumulation at polymer – ceramic interfaces, thus highlighting the crucial role of interfacial dynamics in overall ionic conductivity [320].

**Figure 4.**
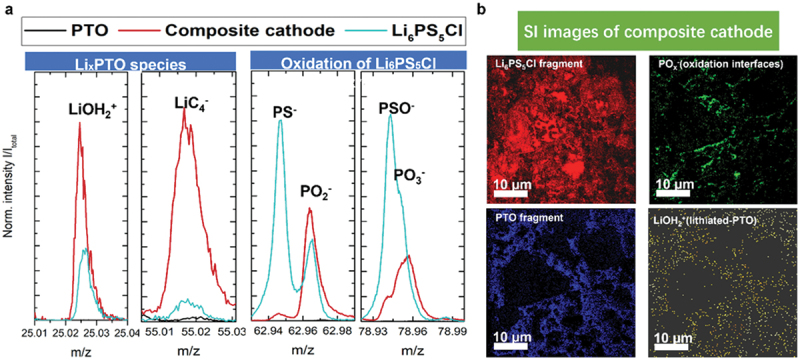
TOF-SIMS analysis reveals the redox interactions between PTO and Li_6_PS_5_Cl. The investigation includes (a) comprehensive mass spectra of pristine pto, Li_6_PS_5_Cl electrolyte, and the composite cathode, and (b) secondary ion images highlighting fragments of Li_6_PS_5_Cl (S^−^, Cl^−^), PO_*x*_^−^ species (PO_2_^−^, PO_3_^−^), PTO-related fragments (C_2_H^−^, C_4_H^−^), and LiOH_2_^+^ within the composite cathode. (reprinted from ref. [295]; copyright (2023) by the American physical Society).

Discussions of SEI and CEI have traditionally centred on liquid-based batteries; however, in recent years, TOF-SIMS has been increasingly applied to visualize and characterize interphases in SSB materials as well [293–296,310]. Although solid electrolytes are generally regarded as chemically stable, interfacial phases often emerge upon contact with active materials, sometimes reducing ionic conductivity but in certain cases enhancing mechanical integrity and cycling stability. For example, Qing Ai et al. examined composite electrodes of argyrodite-type Li_6_PS_5_Cl with pyrene-4,5,9,10-tetraone (PTO) and, using TOF-SIMS, identified spontaneous interfacial redox reactions. The study by Qing Ai et al. investigates the chemical and mechanical interactions at the interface of these materials, emphasizing the effect of lithium-ion transport and its impact on the composite cathode’s structural integrity and electrochemical performance. By employing TOF-SIMS alongside other high-resolution techniques, the authors provide spatially resolved insights into lithium distribution, reaction products, and mechanical properties within the cathode composite. TOF-SIMS revealed spontaneous redox reactions at the PTO/Li_6_PS_5_Cl interface. Lithium ions reacted with PTO, forming Li_*x*_PTO, while concurrent oxidation of Li_6_PS_5_Cl led to the generation of phosphate-based products, such as PO_*x*_^−^ fragments, localized at the interface. The chemical maps showed that these oxidation products accumulated specifically at contact regions, evidencing localized redox activity. Lithium incorporation into PTO was non-uniform, with TOF-SIMS imaging highlighting spatial variations in lithium concentration across the composite (Figure 4). Areas with higher lithium content corresponded to regions of enhanced electrochemical activity. The PTO phases enriched with lithium exhibited enhanced mechanical properties. TOF-SIMS depth profiling correlated higher lithium concentrations with increased Young’s modulus and hardness, indicating that lithium incorporation directly influenced the structural stability of PTO domains. The PTO/Li_6_PS_5_Cl composite cathode displayed a continuous and smooth interfacial boundary, essential for maintaining ion transport pathways and minimizing resistance. This observation was further supported by TOF-SIMS chemical maps, which confirmed the absence of significant voids or delamination at the interface. TOF-SIMS also identified long-term chemical degradation mechanisms, including the formation of secondary products at the interface. These findings suggest that while the PTO/Li_6_PS_5_Cl composite offers initial compatibility, mitigating by-product formation is necessary for prolonged stability. Collectively, using TOF-SIMS, identified spontaneous interfacial redox reactions forming Li_*x*_PTO and phosphate fragments localized at the interface; mapping further revealed non-uniform lithium incorporation correlated with enhanced electrochemical activity and improved mechanical properties, underscoring the importance of spatially resolved interfacial analysis [295].

Similarly, Niaz Ahmad et al. demonstrated that TOF-SIMS depth profiling of a Li_2.96_P_0.98_S_3.92_O_0.06_-Li_3_N glass-ceramic electrolyte revealed a Li_2_O/Li_3_N-rich pre-SEI at the Li interface, suppressing dendrite growth and enabling stable plating/stripping in all-solid-state lithium-metal batteries (ASSLMBs) [293], while Fabian J. Simon et al. applied TOF-SIMS to poly(ethylene oxide)-based polymer electrolytes, identifying a LiF/polysulfide-rich interphase that lowered resistance but continuously thickened with cycling [294]. Extending in SSBs, TOF-SIMS has been applied to Li – S/Se systems, where Chen Zhao et al. showed that highly fluorinated ether electrolytes suppressed Se/S migration, produced a fluorine-rich SEI, and preserved microporous cathode structures [353]. At polymer – metal interfaces, Xu et al. revealed acid-base bonding products and ultra-thin coatings at elastomer/Cr – Al boundaries, while Xu Liu et al. showed via TOF-SIMS that a sulfur-rich, hierarchical SEI stabilized SiO/C anodes at − 20°C by ensuring uniform Li^+^ transport [175,354]. Likewise, TOF-SIMS depth profiling in high-voltage LNMO/graphite cells with polyphenylene sulfide-based separators confirmed suppressed transition-metal migration and HF-induced degradation, improving interfacial stability [355]. In parallel, Feng Jin et al. demonstrated that Li_3_InCl_6_ coatings on NCA cathodes provided uniform coverage, reduced interfacial by-products, and improved lithium diffusion at the NCA/Li_6_PS_5_Cl interface, as evidenced by TOF-SIMS elemental mapping [296]. At higher resolution, Chunli Li et al. used TOF-SIMS to validate a surface fluorinated reconstruction (SFR) mechanism on Ni-rich cathodes, demonstrating dual benefits: stabilized CEI and Ni-induced SEI reinforcement [356]. Complementary work by Nicolas Gauthier et al. revealed cathode-dependent SEI chemistries on Li_4_Ti_5_O_12_ anodes and showed that elevated temperatures or voltages exacerbate manganese dissolution and SEI thickening [288–290]. In lithium-metal systems, TOF-SIMS identified robust, LiF-rich SEIs formed in tetrahydrofuran-based electrolytes [286], phosphorus/nitrogen-containing SEIs from TNNT-modified anodes [309], and diffusion-suppressing Li_3_PO_4_ layers at Li/oxide solid electrolyte interfaces [310]. Related efforts by Niloofar Soltani et al. showed Al_2_O_3_ ALD coatings on Sn-based anodes yielded fluorine-rich SEIs with improved conductivity [174]. Additionally, low-temperature azo reactions were shown to graft radical species onto garnet surfaces, forming a C – F and LiF-rich gradient layer that facilitated uniform Li deposition and enhanced cathode compatibility, as revealed by TOF-SIMS [141]. Wen-Jun Li et al. systematically studied coating layers on lithium metal anodes formed under controlled atmospheres, and SIMS analyses confirmed the formation of Li_2_CO_3_/Li_3_N films with improved interface uniformity [285]. Similarly, Chengwei Ma et al. used TOF-SIMS to reveal dual-layered SEI structures on lithium metal anodes in ether-based electrolytes, showing organic-rich outer layers and inorganic-rich inner layers for ionic conductivity [284]. TOF-SIMS has also clarified Li^+^ transport in SEI films by supporting a two-layer, two-mechanism model, Fickian diffusion in a porous organic layer and a knock-off mechanism in dense Li_2_CO_3_ domains – providing fundamental insight into SEI ion transport [271]. Likewise, TOF-SIMS monitoring of halide-based electrolytes revealed that while Li_2.6_Zr_0.4_Ho_0.6_Cl_6_ degraded at high voltages, it formed stable interphases protecting Ni-rich cathodes [209]. Thus, with the help of TOF-SIMS, it is widely recognized that in all types of batteries, including LIBs with liquid, solid, or polymer electrolytes, the anode is consistently covered by a thin SEI [266,277], comprising LiF, Li_2_CO_3_, LiCO_2_-R, Li_2_O, lithium alkoxides, and sometimes nonconductive polymers [268,278–283].

Further, transition metal-based electrodes have also been extensively studied using TOF-SIMS to elucidate interfacial processes and SEI/CEI evolution across diverse systems. In chromium-based systems, Jun-Tao Li et al. employed TOF-SIMS to track Cr^+^ and CrCs^+^ ions on ultrathin Cr_2_O_3_ films, revealing a converted outer Li_2_O-rich layer and an unconverted inner Cr_2_O_3_ region, with reduced Cr accumulating at the interface to form a dense barrier that hinders lithium transport [185]. SIMS analysis on electroplated Sn – Co films further identified SEI formation, surface oxide conversion, and cracking of the delithiated alloy layer, highlighting changes in C, Li, and Cu ion distributions [186]. Similarly, TOF-SIMS depth profiling of Sn – Ni alloy electrodes revealed incomplete initial lithiation, partitioning the alloy into fully and partially lithiated regions, with subsequent cycling causing island-like structures filled by SEI, trapping lithium and chlorine while preserving active material [348]. In NiO thin films, TOF-SIMS identified early nickel migration into the SEI and lithium accumulation at the Ni/NiO interface, driving nanoparticle nucleation and surface roughening, emphasizing interface dynamics in conversion reactions [349]. Investigations of transition-metal crossover in lithium-metal batteries using TOF-SIMS demonstrated heterogeneous Ni and Mn deposition, correlated with thick, uneven SEI layers in standard LP57 electrolytes, while high-voltage electrolytes reduced metal deposition and enhanced SEI uniformity, underscoring the impact of electrolyte composition on interfacial stability and dendrite mitigation [350]. In copper-based electrodes, TOF-SIMS revealed TiO_2_ distribution throughout Cu_*x*_O matrices, influencing SEI formation and lithium/electron transport [190], while studies on lithiated a-Si/Cu interfaces showed Li segregation at the interface, reducing adhesion but enabling near-frictionless sliding, and SEI ‘breathing’ behaviour during cycling was tracked via dynamic changes in lithium-containing compounds [351,352]. Iron-based electrodes similarly benefited from TOF-SIMS analysis, which elucidated incomplete conversion/deconversion of FeS and Fe_2_O_3_ thin films, SEI composition fluctuations, penetration of decomposition products, and lithium diffusion heterogeneity across outer Li-rich, transitional, and inner Li-trace regions, highlighting the performance-limiting role of the SEI and converted material [157,200,344–346]. In LiFePO_4_ cathodes, TOF-SIMS revealed phosphorus- and nitrogen-rich CEI layers that enhanced interfacial stability and durability [207]. Finally, cobalt-based systems were probed to study SEI/CEI formation on graphite and LiCoO_2_ electrodes in PC-based electrolytes, revealing sulphur and phosphorus species distributions, interfacial Co reduction in LiPON/LiCoO_2_ thin films, and correlations between conductive binder distribution and CEI formation in thick electrodes, while helium ion microscopy combined with in situ TOF-SIMS on LiCoPO_4_ highlighted uneven CEI growth, cobalt dissolution, and lithium accumulation as key factors in high-voltage cathode instability [56,287,357,358].

Silicon- and carbon-based electrode systems, including graphite, Si, Si@C composites, and MXene-modified interfaces, have been extensively studied for SEI formation and evolution using TOF-SIMS, too. Such as Catarina Pereira-Nabais et al. demonstrated that silicon electrode morphology, such as amorphous hydrogenated silicon (a-Si:H) and silicon nanowires (SiNWs), strongly influences SEI chemistry, showing two-step lithium profiles with organic and inorganic components and highlighting the role of LiCl, LiF, and electrolyte-derived residues in the outer SEI [327], while subsequent studies observed SEI volume changes during lithiation/delithiation, with lithium entrapment limiting ion transport and reducing the apparent diffusion coefficient (~5.9 × 10^−10^ cm^2^/s) [336]. Sn coatings on SiNWs were shown by Kohandehghan et al. to enhance coulombic efficiency and cycling retention, with TOF-SIMS revealing intensified lithium signals near current collectors due to interface SEI formation [337]. Nakai et al. identified distinct SEI compositions depending on electrolyte, with FEC-derived layers providing LiF and polyene compounds that protect against electrode oxidation [338], while Schroder et al. emphasized the heterogeneous SEI on silicon surfaces and the influence of native oxides, using TOF-SIMS depth profiling combined with multivariate analysis to resolve organic-inorganic stratification and surface chemistry effects [339]. Bordes et al. confirmed two-phase lithiation fronts and SEI thickening in amorphous Si thin films [340], whereas Kumar et al. highlighted strain-induced SEI thickening and lithium loss in patterned Si electrodes [341]. Stetson et al. and Wu et al. demonstrated SEI heterogeneity and stability differences between binder-free Si@C-network and binder-containing Si electrodes, showing that TOF-SIMS depth profiling correlates with lithium incorporation, layer cohesion, and electrochemical performance [55,342]. Nguyen et al. revealed that prelithiation using SLMP increases inorganic LiF content, improving SEI uniformity and lithium-ion transport [343], while Xu et al. showed that HF-etched Si surfaces produce thicker, Li-trapping SEIs compared to native-oxide Si, affecting lithiation reversibility [328]. Berthault et al. used lithium isotope tracing with TOF-SIMS to elucidate rapid surface-dominated lithium exchange followed by slower diffusion into the bulk, revealing mobility differences among silicide phases [329]. Zhang et al. optimized SiO/C composite anodes through prelithiation and electrolyte selection, with LiTFSI-based SEIs showing improved ionic conductivity, mechanical stability, and reduced volume expansion [330].

In carbon-based systems, Peled et al. provided the first direct confirmation of polymeric SEI components on HOPG with TOF-SIMS, showing plane-dependent functionality and compositional heterogeneity [331], while Watkins et al. and Kubota et al. examined interfacial reactions and additive-induced SEI formation on graphite, highlighting organo-fluorine and LiF-rich layers that improve cycle performance [332,333]. Okumura et al. demonstrated DMAC-induced SEI polymerization and thermal stability enhancements [334], and Padwal et al. showed glyme-based electrolytes form bilayered, stable SEIs on SiO_*x*_/C anodes, minimizing cracking and maintaining ionic conductivity [335]. Carbon-based SIMS studies also revealed stress formation near graphite surfaces and air-exposure effects on SEI composition [321,322], while MALDI-TOF MS confirmed polymer-rich SEIs with dynamic structural evolution [323]. Ma et al. and Ho et al. revealed chemical composition and lithium exchange dynamics in Sn/graphene and graphite anodes [324,325], and Xu et al. demonstrated Ni^2+^ induced SEI thickening and increased resistance in Ni-rich systems [326]. Fluorinated graphene additives enhanced LiF-rich SEIs for dendrite suppression and uniform Li-ion transport [311]. In MXene- and peptide-modified solid-state systems, Zhao et al. and Zhang et al. confirmed in situ SEI generation, selective LiF/Li_3_N distribution, and improved ionic conductivity through TOF-SIMS 3D imaging [315,316], while Haridas et al. highlighted additive-induced interface stabilization in Si anodes and NMC cathodes [292]. Finally, TOF-SIMS studies on conventional LIBs by Lee et al. and Dupre et al. consistently reveal SEI inhomogeneity, lithium depletion, and the critical role of interphase chemistry in long-term cycling stability [359,360].

Building on these insights into carbonaceous and silicon-based anodes, attention turns to ionic liquid, hybrid interstitial layers, complex lithium additive and salts. Ye Zhu et al. demonstrated that LiDFOB forms a thinner yet more robust SEI on graphite anodes while mitigating lithium trapping caused by transition metal migration from Li_1.2_Ni_0.15_Mn_0.55_Co_0.1_O_2_ cathodes, as revealed by SIMS analysis, with sacrificial oxidation at the positive electrode forming a passivation layer that reduces impedance and suppresses metal dissolution [299]. In a similar vein, Dongni Zhao et al. showed that LiDFBOP produces a layered SEI with organic-rich outer layers and LiF-dominated inner layers, improving lithium-ion transport, cycling stability, and reducing electrolyte salt degradation; when applied to LiNi_0.5_Mn_1.5_O_4_ cathodes, it forms dense, inorganic-rich CEI films that enhance lithium mobility and rate capability [300,301]. Weimin Zhao et al. further confirmed, using TOF-SIMS and ion chromatography, that LiDFBOP creates uniform, LiF- and phosphate-rich interfacial layers on NMC622 cathodes and SiO-C anodes, suppressing side reactions and transition metal dissolution [302]. TriMethylene Sulfite has been shown to promote thin, uniform, LiF- and sulfur-enriched SEI layers on graphite anodes, enhancing lithium-ion transport and capacity retention [303], while pyridinium salts decompose at ~2.5 V to yield robust 2-methylpyridinium-enriched SEI that stabilizes high-voltage cathode interfaces and suppresses gas evolution [304]. Ang Fu et al. used DMMA to form compact, inorganic-enriched CEI on LiCoO_2_ cathodes, limiting cobalt dissolution and reducing electrolyte degradation, as revealed by TOF-SIMS depth profiling [305], and triphenylphosphine selenide similarly stabilizes SEI and CEI layers through multifunctional interfacial engineering [306]. FEC addition to aqueous LiTFSI electrolytes modifies TFSI^−^ solvation, leading to thinner CEI layers and enhanced ion transport, as confirmed by TOF-SIMS, SEM, and Raman imaging [307], while comparative studies show EC-, PC-, and FEC-based electrolytes form distinct CEI compositions, with FEC generating LiF-rich, uniform layers that improve oxidative stability [361]. Kjell Schroder et al. demonstrated that FEC produces thicker (~35.1 nm), inorganic-rich SEI with improved cycling efficiency compared to conventional electrolytes (~23.1 nm) [308]. Super-concentrated ionic liquid electrolytes facilitate the formation of thin, LiF-rich SEI and uniform lithium nucleation at high current densities, suppressing dendrite growth, as revealed by TOF-SIMS [312], while the SEI thickness, predominantly LiF, was identified as the key determinant of Coulombic efficiency in gas-saturated ionic liquid electrolytes [313]. In magnesium alloys, TOF-SIMS showed that multi-layered films formed by ionic liquid components suppress hydrogen evolution and water penetration [314]. Hybrid interfacial layers combining nano silica aerogel and poly-methacrylate generate biphasic SEI with Li – Si alloy and LiF, confirmed by TOF-SIMS and SEM, offering high ionic conductivity, dendrite suppression, and enhanced cycling stability [317]. In PEO-based solid polymer electrolytes, phosphorus pentasulfide promotes Li_2_O-rich SEI with uniform horizontal and vertical composition, improving lithium-ion transport and minimizing degradation, as revealed by TOF-SIMS 3D imaging [318]. Conversely, vinylethylene carbonate forms ineffective anode SEI but generates a robust CEI via electro-polymerization, with TOF-SIMS confirming deeper lithium penetration and cross-talk-mediated CEI formation on NMC622 cathodes [319]. Finally, MUI-derived CEIs minimize electrolyte decomposition [362], and TOF-SIMS-based studies have linked distinct CEI chemistries to degradation pathways in Ni-rich, spinel, and Li-rich cathodes [363]. Collectively, these studies highlight that TOF-SIMS is indispensable for elucidating SEI/CEI composition, thickness, and distribution, providing mechanistic insight and guiding the rational design of additives and interfacial layers for enhanced battery performance.

**Figure 5.**
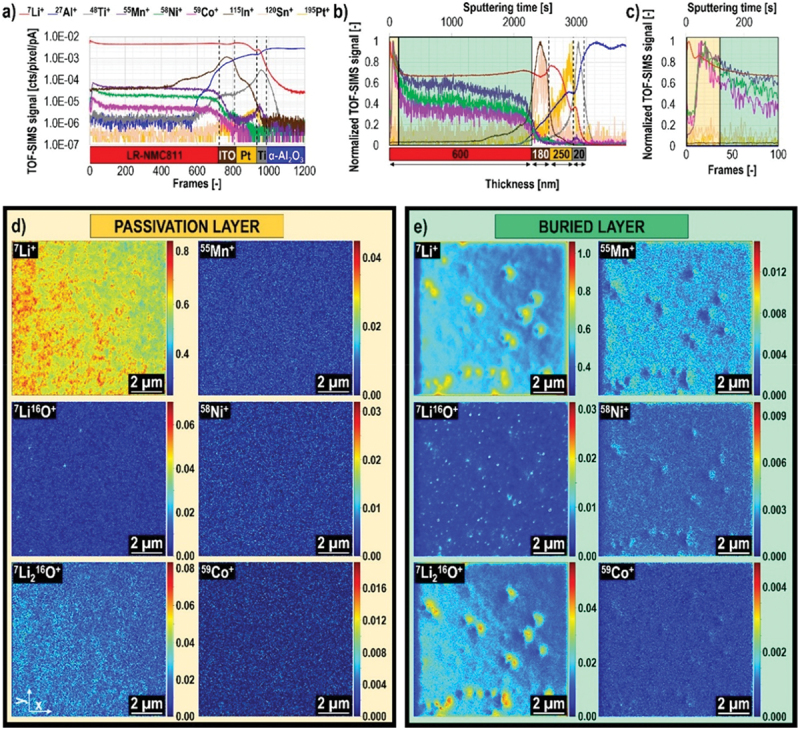
The chemical structure of the LR-NMC811/ITO/Pt/Ti/α-Al_2_O_3_ system after 30 days of air exposure is analyzed. (a) Depth profiles display the primary components across the sample. (b) Depth profiles are normalized to unity, with Black dashed lines marking the interfaces between layers, while beige and green rectangles denote the approximate locations of the passivation and buried layers, respectively. (c) A detailed view highlights the depth profiles near the passivation layer. (d) Two-dimensional chemical maps represent the passivation layer, averaging signals across frames 0–59. (e) Similar 2D chemical maps illustrate the buried layer, with signal averaging conducted over frames 60–922. This multi-faceted analysis provides insights into the chemical evolution of the structure upon prolonged air exposure. (reprinted from ref. [54]; copyright (2023) by the American physical Society).

Furthermore, TOF-SIMS has proven invaluable in advancing battery research by providing detailed insights into degradation mechanisms and interfacial phenomena [54,79,80,244,324,334,346,364–396]. Its ability to generate high-resolution mass spectra enables the identification of degradation products. Typically, degradation analysis is conducted using ex-situ measurements by comparing samples before and after charge – discharge cycling tests. For example, Agnieszka Priebe et al. provided critical insights into the microstructural and chemical evolution of Li-rich NMC811 thin films, revealing the presence of 400 ± 100 nm overlithiated grains and 100 ± 30 nm nanoparticles enriched in lithium species using TOF-SIMS [54]. These lithium-rich domains, which may serve as reservoirs to counteract lithium loss during battery operation, undergo significant decomposition under ambient conditions, leading to a homogeneous elemental distribution and surface passivation by Li_2_CO_3_ and LiOH. The high reactivity of Li in NMC811 leads to rapid formation of a passivation layer upon brief air exposure, significantly altering the surface chemical structure. To study this evolution, a sample was stored under ambient conditions for 30 days. Depth profiling revealed the coexistence of a surface passivation layer and a buried Li_X_Ni_0.8_Mn_0.1_Co_0.1_O_2_/NMC811 (LR-NMC811) region (shown in Figure 5).

While the buried layer maintained consistent ion signals (e.g. ^7^Li^+^, ^55^Mn^+^, ^58^Ni^+^, ^59^Co^+^), the passivation layer exhibited substantial chemical changes. Signals for transition metal ions diminished, likely due to surface oxidation and reduced ionization efficiency, as shown in Figure 5. These findings align with reports of carbonate and hydroxy species, such as Li_2_CO_3_ and LiOH, forming on NMC811 surfaces [369]. Sputtering times for the passivation layer and buried region increased by factors of ~1.5 and ~1.3, respectively, in aged samples, suggesting structural changes. Two-dimensional chemical maps showed irregular islands of enhanced ^7^Li^+^ and ^7^Li^16^O^+^ signals in the aged passivation layer, contrasting with the uniform distributions observed in fresh samples. These distributions did not correlate, implying a complementary relationship between Li and O. The buried layer remained chemically homogeneous, with no evidence of over-lithiated grains or nanoparticles as seen in fresh samples. The increased ^7^Li^16^O^+^ (1.4×) and ^7^Li^12^C^+^ (5×) signals in aged samples suggest the progressive replacement of LiOH by Li_2_CO_3_, a hypothesis supported by reduced ^1^H^7^Li^+^ signals. This transition indicates time-dependent chemical stabilization on the surface. While TOF-SIMS data confirmed these changes, limitations in detecting negatively charged ions, such as CO_3_^2−^ and OH^−^, necessitate further analysis using complementary techniques, such as FTIR spectroscopy. While the depth profiling comparisons between fresh and aged samples under consistent experimental conditions revealed a conserved number of secondary ions in the buried layer, suggesting that the passivation layer protects against contamination by H, C, N, and O. However, changes in spatial atom/molecule rearrangement appear to minimally affect the deeper chemical structure.

**Figure 6.**
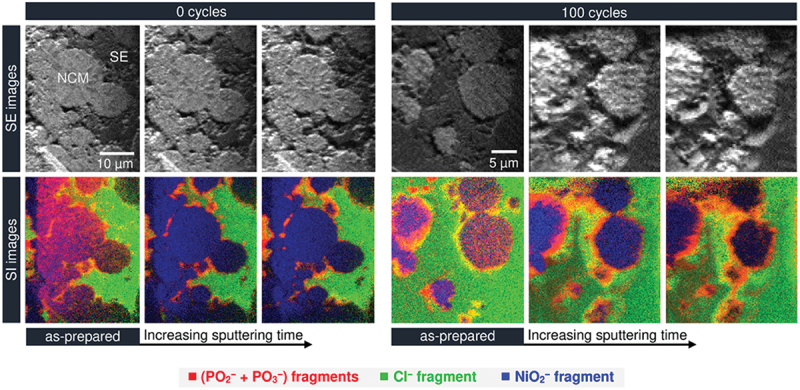
TOF-SIMS was employed to analyse crater sidewalls of cathodes before and after cycling. Secondary electron (SE) and secondary ion (SI) images of the crater sidewalls were obtained as a function of sputtering time, which also served as a surface cleaning step. This cleaning process, necessary to remove defect layers introduced during fib sample preparation, restored the original signals by gently sputtering the surface using a rasterized analysis beam in direct current (DC) mode. si imaging revealed a notable increase in PO_*x*_^−^ and SO_*x*_^−^ fragments, particularly at the NMC622/SE interface, which can be attributed to battery cycling, as detailed in the supporting information. These fragments facilitated imaging of the sei. Furthermore, the distinct sputtering rates of NMC622 and se enabled the selective excavation of particles. (reprinted from ref. [79]; copyright (2019) by the American physical Society).

Moreover, such degradation analyses have become feasible even for SSBs due to advancements in cross-sectional preparation techniques. For example, Felix Walther and colleagues conducted a detailed investigation into the interfacial degradation mechanisms in SSBs with composite cathodes utilizing Li_6_PS_5_Cl solid electrolytes and NMC622 cathode materials [79]. Their study focused on characterizing the SEI, which plays a critical role in battery performance and durability, using advanced analytical techniques such as TOF-SIMS. TOF-SIMS revealed that the SEI predominantly comprises phosphate (PO_*x*_) and sulphate (SO_*x*_) species, forming a uniform layer around the NMC622 particles, with these compounds becoming more pronounced during battery cycling, as shown in Figure 6. Transition-metal sulfide, phosphides, and chlorides were found to have a minor role in SEI formation, as their signals diminished after cycling. Surface and depth profiling localized phosphate and sulphate species at the NMC622/SEI, with notable enrichment after cycling. The SEI, formed through interfacial reactions involving oxygen from NMC622 and phosphorus and sulphur from Li_6_PS_5_Cl, was estimated to be less than 10 nm thick, with its composition and morphology significantly influencing battery cycling stability. These findings highlight the importance of understanding SEI formation for improving cathode coatings and additives, thereby mitigating degradation and enhancing battery life. While the research by Felix Walther et al. also investigates the effects of vapor-grown carbon fibres on degradation pathways in composite cathodes of lithium thiophosphate-based SSBs [383]. Advanced analyses using TOF-SIMS and XPS revealed that vapor-grown carbon fibres enhance initial capacity by improving active material utilization and supporting redox-active reactions. However, its inclusion also accelerates degradation through the formation of long-chain polysulfides and localized phosphate species at cathode interfaces. The study identifies decomposition mechanisms at the vapor-grown carbon fibres/SEI as critical contributors to capacity fading, providing valuable insights for the development of protective strategies to improve the durability and performance of SSBs.

**Figure 7.**
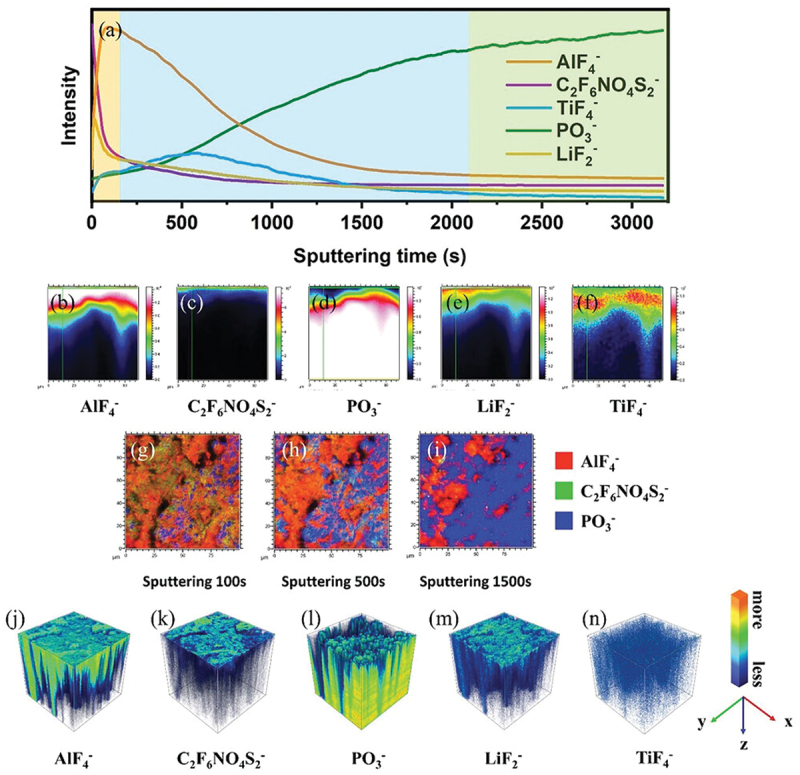
Illustrates TOF-SIMS analysis in negative ion mode, highlighting the chemical composition and distribution on and near the LATP pellet surface after cycling. Panel (a) presents in-depth profiles of secondary ion fragments AlF_4_^−^, C_2_F_6_NO_4_S_2_^−^, PO_3_^−^, LiF_2_^−^, and TiF_4_^−^, revealing the surface and subsurface distribution of AlF_3_ and related components. Panels (b– f) provide side-view maps of fragments mentioned above, showing their spatial distribution at varying depths. Panels (g–i) display chemical maps of AlF_4_^−^, C_2_F_6_NO_4_S_2_^−^, and PO_3_^−^, as a function of sputtering depth. Finally, panels (j–n) present a 3D visualization of the spatial distribution of same fragments within the sputtered volume, offering insights into the electrofusion process. (reprinted from ref. [80]; copyright (2023) by the John Wiley and sons).

Furthermore, Yuping Gu et al. explored strategies to stabilize and optimize NASICON-based solid-state lithium-fluoride conversion batteries, addressing interfacial challenges between the Li metal anode and Li_(1+*x*)_Al_*x*_Ti_(2−*x*)_(PO_4_)_3_ (LATP) solid electrolyte [80]. Their study introduced a high-surface-area AlF_3_ (HS-AlF_3_) interlayer as a novel interface fluorination strategy to improve compatibility, stability, and performance at the LATP/Li interface. TOF-SIMS was used to investigate the evolution of the LATP surface coated with an AlF_3_ layer during electrochemical cycling. LATP electrolytes were extracted from coin cells after 50 cycles at 100 µA cm^−2^, polished to remove residual AlF_3_, and analyzed in regions with a thin AlF_3_ layer. Figure 10 displays the depth profiles and 3D reconstructions of sputtered volumes for species of interest: AlF_4_^−^, C_2_F_6_NO_4_S_2_^−^, PO_3_^−^, LiF_2_^−^, and TiF_4_^−^, representing AlF_3_, interface agents, LATP electrolyte, and newly formed cycling products. High AlF_4_^−^ and C_2_F_6_NO_4_S_2_^−^ signals at the initial sputtering stages (Figure 7a) confirmed the presence of an AlF_3_ layer with TFSI^−^ from interface agents. Elevated LiF_2_^−^ signals suggested LiF formation via TFSI^−^ decomposition, which enhances interfacial stability and compatibility between the Li anode and LATP electrolyte [387]. In contrast, PO_3_^−^ intensity was initially weak but increased throughout sputtering, indicating a transition from the AlF_3_ layer to the LATP electrolyte [386]. A TiF_4_^−^ layer was observed at the AlF_3_-LATP interface, likely formed through fluorination reactions. This fluoride-rich TiF_4_^−^ layer acts as a trap for Ti^4+^, suppressing its reduction to Ti^4+^ and mitigating redox activity, thereby improving electrolyte stability [388]. The mesoporous structure of AlF_3_, with its acidic and unsaturated aluminium sites, facilitates partial dissolution in IA (LiTFSI/acetonitrile +1,1,2,2-tetrafluoroethyl-2,2,2-trifluoroethyl ether).

Dissolved fluorine species diffuse and fluorinate the LATP surface, forming Ti-F and TiF_4_ species with stronger ionic bonds compared to Ti-O or Ti-(PO_4_) bonds. The relatively porous AlF_3_ interlayer buffers volume changes during trap layer formation, enhancing interfacial stability. Side-view maps (Figure 7b–f) and RGB overlap mappings (Figure 7g–i) illustrate the compositional evolution during sputtering, with a shift from AlF_3_ to LATP as sputtering progresses [80]. After 100 s, AlF_3_ dominates, while after 1500 s, LATP becomes prevalent as PO_3_^−^ intensities rise and AlF_4_^−^ diminishes. The 3D reconstructions (Figure 7j–n) offer detailed insights into species distributions, highlighting the penetration of AlF_3_ decoration and the fluorine-rich trap zone stabilizing LATP and Li anode interfaces. TOF-SIMS analysis revealed the in-situ formation of a fluorine-rich interlayer, predominantly TiF_4_, at the LATP/AlF_3_ interface, acting as a protective barrier against Ti^4 +^ reduction to Ti^3 +^. This fluorine-rich layer, enriched with LiF and TiF_4_, minimized redox activity and structural degradation of LATP, while facilitating the formation of LiF domains to promote efficient Li-ion conduction and reduce interfacial resistance. Consequently, the Li-ion transference number was significantly enhanced, supporting sustained electrochemical performance. Furthermore, the fluorinated interlayer suppressed lithium dendrite formation, maintaining a stable interface over extended cycling periods. Depth profiling by TOF-SIMS demonstrated a gradual and homogeneous transition from the AlF_3_ interlayer to the LATP substrate, with minimal degradation and preserved porous structure of the AlF_3_ layer to buffer volume changes. These advancements enabled NASICON-based batteries with AlF_3_ interlayers to exhibit remarkable stability, maintaining over 500 hours of cycling with reduced overpotential in symmetric Li||Li cells. Full cells employing LiFePO_4_ and FeF_3_ cathodes achieved excellent capacities, reaching up to 696.7 mAh g^−1^ for FeF_3_, with superior cycling stability. TOF-SIMS further confirmed the preservation of interfacial integrity throughout prolonged cycling, solidifying the effectiveness of the fluorination strategy in enhancing battery performance.

## 4. Isotope diffusion experiments for battery materials

In SIMS analysis, the ionization probability varies depending on the material, making quantitative analysis challenging. However, since isotopes exhibit identical chemical properties, SIMS allows for accurate quantitative analysis of isotope ratios. Therefore, by SIMS measuring the distribution of isotopes within a sample, diffusion coefficients can be determined. In battery materials, the diffusion coefficient of the mobile ion helps to elucidate the ion transport mechanisms and kinetic limitations of battery performance. The diffusion coefficient, *D*, is known to follow the Arrhenius law, which can be expressed by the following equation when the activation energy and the pre-exponential factor are denoted as *E*_a_ and *D*₀, respectively:

(2) $$\begin{array}{c} D=D_{0}e^{-\frac{E_{a}}{k_{B}T}}. \end{array}$$

Here, *k*_B_ is the Boltzmann constant, and *T* is the absolute temperature. The activation energy of *D* reflects the energy barrier for ion hopping and typically coincides with the activation energy obtained from the temperature dependence of ionic conductivity.

To determine lithium diffusivity, samples are typically prepared through isotope exchange. With a natural abundance of ^7^Li:^6^Li = 92.5:7.5, isotope exchange can be applied by joining a ^6^Li-enriched material with one of natural abundance. Alternatively, isotope exchange methods employing ^6^Li metal foils or ^6^Li-enriched liquid electrolytes may be used, with the choice determined by the physical and chemical properties of the material. The analysis of SIMS profiles is performed using conventional diffusion equations to evaluate the diffusion coefficient [110,398]. In a diffusion couple of the same material with different isotopic concentrations, the solution to Fick’s second law for one-dimensional diffusion in a semi-infinite medium is given by:

(3) $$\begin{array}{c} \frac{C\left(x,t\right)-C_{0}}{C_{1}-C_{0}}=\frac{1}{2}\left[1-\mathrm{erf}\left(\frac{x}{2\sqrt{D^{*}t}}\right)\right], \end{array}$$

provided that the diffusion length is shorter than the sample thickness and the diffusion coefficient is isotope-independent. Here *C*(*x*, *t*) is the concentration at distance *x*, *C*_0_ and *C*_1_ are initial concentrations in the respective regions, D^*^ is the tracer diffusion coefficient, and *t* is the diffusion time, which is often substituted by the annealing time in experiments. Using SIMS isotope profiles of ^6^Li/(^7^Li + ^6^Li), *D* values are extracted through curve fitting.

**Figure 8.**
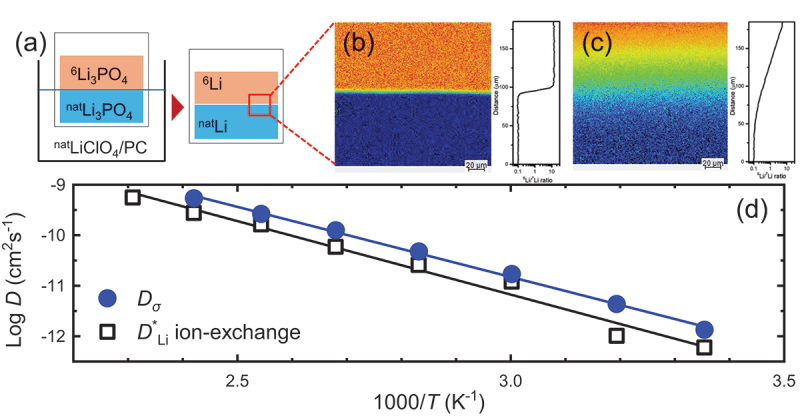
(a) Schematic diagram of sample preparation via ion exchange method. Isotope mappings of ^6^Li/^7^Li for amorphous Li_3_PO_4_ thin films; (b) as prepared and (c) annealed at 120°C for 24 h. (d) Temperature dependence of D* and *D*_*σ*_ for amorphous Li_3_PO_4_ thin films. (reprinted from ref. [202]; copyright (2016) by the elsevier).

As an example, ^6^Li isotope tracer measurements in amorphous Li_3_PO_4_ thin films are presented [202]. The natural abundance ratio of lithium is ^7^Li:^6^Li = 92.5:7.5. As shown in Figure 8a, Kuwata et al. prepared a diffusion couple by performing ion exchange through the immersion of a ^6^Li-enriched thin film in a liquid electrolyte with natural isotopic abundance of lithium. Figure 8b shows the ^6^Li isotope distribution immediately after ion exchange, exhibiting a step-like profile. After annealing at 120°C for 24 hours, the distribution changes to a gradual slope due to diffusion, as shown in Figure 8c. By fitting the isotope distribution obtained from SIMS to Equation (2), diffusion coefficients at various annealing temperatures were determined (Figure 8d). *D*_*σ*_ represents the conductivity diffusion coefficient, calculated from the ionic conductivity, *σ*, and the number density of ions, *n*. According to the Nernst – Einstein equation [398], can be expressed as:

(4) $$\begin{array}{c} D_{\sigma }=\frac{k_{B}T}{n{\left(\mathrm{ze}\right)}^{2}}\sigma , \end{array}$$

where *z* is the valence of the ion (1 for Li^+^) and *e* is the elementary charge. At room temperature (25 °C), D* was experimentally obtained to be 6.0 × 10^−13^ cm^2^s^−1^. The activation energy of D*was evaluated to be 0.58 eV, which was in close agreement with that of *D*_*σ*_, 0.55 eV. A considerable difference is observed between D* and *D*_*σ*_, as *D*_*σ*_ is greater than D* over the entire temperature range. Notably, D* and *D*_*σ*_ are related by the Haven ratio as follows:

(5) $$\begin{array}{c} H_{R}\;\equiv \;\frac{D^{*}}{D_{\sigma }}. \end{array}$$

The Haven ratio, *H*_R_, is a correction factor of the Nernst-Einstein equation, caused by the correlated charge transport [399,400]. When *H*_R_ = 1, individual independent charge transport can be assumed. *H*_R_ of the amorphous Li_3_PO_4_ thin films was 0.55. The small value of *H*_R_ ( <1) indicated the correlative motion of lithium-ions in the amorphous Li_3_PO_4_.

**Figure 9.**
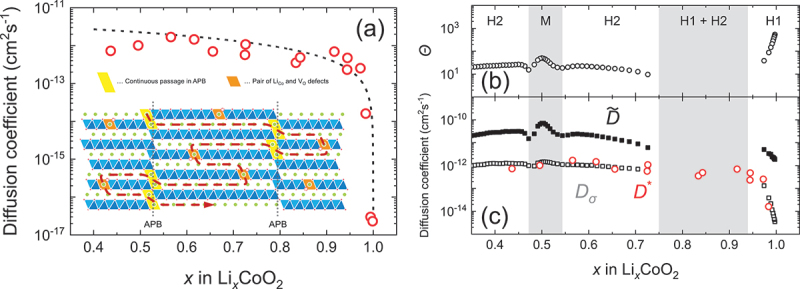
(a) Lithium composition *x* dependence of D* for lco thin film and schematic model of *c*-axis diffusion in the lco thin films. Li composition *x* dependence of *Θ* (b), $\tilde{D}$, and *D*_*σ*_ (c) for Li_*x*_CoO_2_ thin films measured by pitt. (reprinted from ref. [133]; copyright (2021) by the Royal Society of chemistry).

Isotope diffusion measurements using SIMS can, in principle, be applied to a wide variety of ion conductors by tailoring the isotope source. Examples include solid electrolytes such as LLZTO [136,205] and LLTO [201,401], as well as polymer electrolytes [210]; cathode materials like LMO [111,126,397,402], LCO [133,403], and NMC [103,108,109]; anode materials like silicon [404,405]; and coating materials for active materials [131]. This approach is particularly valuable for mixed ionic – electronic conductors, such as active materials, where conventional conductivity measurements are difficult to perform. An illustrative example is the study of the isotope diffusion along *c*-axis in Li_*x*_CoO_2_ thin films, conducted by Hasegawa and Kuwata et al. [133]. By applying a step-isotope-exchange method, D^*^ was determined across varying lithium compositions (0.4 < *x* < 1.0), with values ranging from 10^−17^ to 10^−12^ cm^2^s^−1^. Near stoichiometric composition, D^*^ decreases drastically, explained by the vacancy diffusion mechanism, where diffusion is proportional to lithium vacancies, as shown in Figure 9a. Based on the observation that the D* along the *c*-axis varies proportionally with the bulk vacancy concentration, a new diffusion model along the *c*-axis was proposed, as illustrated schematically in Figure 9a. This model assumes that lithium-ions diffuse through Co-Li antisite defects and antiphase boundaries. For long-range transport along the *c*-axis, lithium-ions must migrate laterally in the *ab*-plane from one defect to another. Since the diffusion path is significantly longer than the direct *c*-axis distance, the resulting diffusion coefficient is much smaller than that of direct penetration.

Additionally, Hasegawa et al. compared the diffusion coefficient obtained electrochemically with the D*. The chemical diffusion coefficient, $\tilde{D}$, was determined from current relaxation curves using the potentiostatic intermittent titration technique (PITT) [133]. $\tilde{D}$ is used when a lithium concentration gradient is formed within the active material. In operating batteries, lithium transport is directly governed by $\tilde{D}$. $\tilde{D}$ can be expressed in terms of *D*_*σ*_ as follows:

(6) $$\begin{array}{c} \tilde{D}=\Theta D_{\sigma }. \end{array}$$

Here, *Θ* is the thermodynamic factor, which is defined as:

(7) $$\begin{array}{c} \Theta =-\frac{eC_{\mathrm{Li}}}{k_{B}T}\frac{dE}{dC_{\mathrm{Li}}}, \end{array}$$

where *C*_Li_ is the lithium composition and, *E* is the electrode potential. The lithium composition dependence of *Θ* and $\tilde{D}$ is shown in Figure 9b, c, respectively. *D*_*σ*_ was found to be consistent with D*. In contrast, $\tilde{D}$ does not decrease significantly near the stoichiometric composition, since *Θ* increases in a way that offsets the decline in *D*_*σ*_. This enables the battery to operate even near the stoichiometric composition, where D* decreases sharply.

Moreover, interfaces are critical in ion transport. For example, when the surface exchange rate between the isotope source and the sample is limited, isotope profiles are affected by interfacial kinetics, which can potentially lead to inaccurate diffusion coefficients. This can be accounted for using a one-dimensional diffusion solution for a semi-infinite medium with finite surface exchange:

(8) $$\begin{array}{rl} & \frac{C\left(x,t\right)-C_{0}}{C_{s}-C_{0}}=\mathrm{erfc}\left(\frac{x}{2\sqrt{\mathrm{Dt}}}\right)\\ & -\exp\left(\mathrm{hx}+h^{2}\mathrm{Dt}\right)\mathrm{erfc}\left(\frac{x}{2\sqrt{\mathrm{Dt}}}+h\sqrt{\mathrm{Dt}}\right), \end{array}$$

where *C*_0_ is the initial concentration within the sample, and *C*_s_ is the concentration of the external isotope source, assumed to be constant due to its excess amount. The parameter *h* is a constant defined as *h* = *k*/*D*, where *k* is the surface exchange rate and *D* is the diffusion coefficient of the sample. This model is particularly useful for analysing cathode materials near stoichiometric compositions, where interfacial resistance is high [111,133,402].

**Figure 10.**
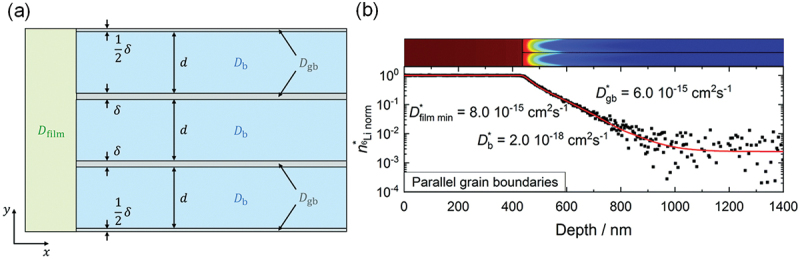
(a) Schematic illustration of the parallel grain boundary diffusion model (Fisher model). (b) Measured (Black squares) and simulated (red line) diffusion profiles in the LiMn_2_O_4_ pellet. (reprinted from ref. [397]; copyright (2019) by the Royal Society of chemistry).

Furthermore, grain boundaries are also essential interfaces in solid materials. Theoretical analysis of isotope diffusion across grain boundaries is often based on the Fisher model, which assumes that grain boundary diffusion occurs at a rate faster than bulk diffusion, as shown in Figure 10a. As supported by the general solutions for Fisher model [406,407], it is empirically known that the grain boundary product, *δD*_gb_, can be calculated from the slope of the linear second part of ln(*C*) *vs. x*^6/5^ [112]:

(9) $$\begin{array}{c} \delta D_{\mathrm{gb}}=1.322{\left(\frac{D_{\mathrm{bulk}}}{t}\right)}^{12}{\left(-\frac{\partial \ln C}{\partial x^{\frac{6}{5}}}\right)}^{-\frac{5}{3}}, \end{array}$$

where *x* is the diffusion depth, *D*_bulk_ is the bauk diffusion coefficient, *D*_gb_ is the grain boundary diffusion coefficient, and *δ* is the width of the grain boundary. In the case of LMO cathodes, Okumura et al. analysed grain boundary diffusion using Equation (9) [111]. More recently, direct calculation of isotope distribution has become feasible. Schwab et al. evaluated grain boundary diffusion in LMO pellets using numerical methods, as shown in Figure 10b [397]. Based on the results of Kuwata et al. on the lithium composition dependence of diffusion coefficients in LMO thin films [402], it was shown that near the stoichiometric composition, *D*_gb_ is greater than *D*_bulk_, whereas in the delithiation phase, *D*_bulk_ exceeds *D*_gb_.

**Figure 11.**
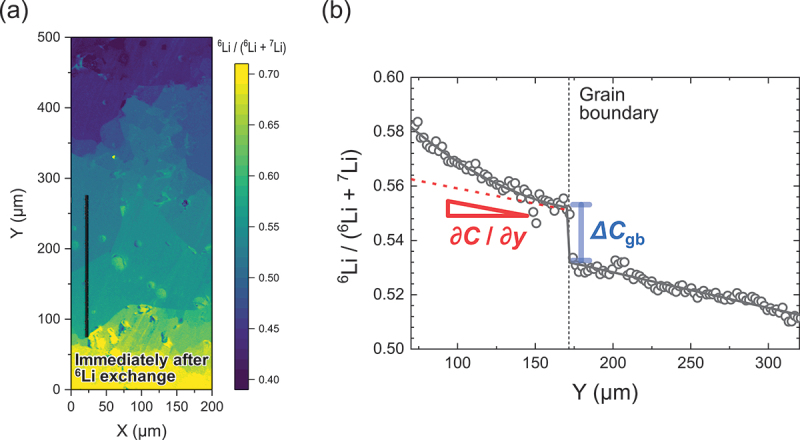
(a) Imaging of the relative ^6^Li fraction of llto immediately after ^6^Li isotope exchange for 59 h. (b) ^6^Li isotopic profiles of the Black line shown in the sims image. (reprinted from ref. Reference [401]; copyright (2021) by the Royal Society of chemistry).

In materials with high bulk ionic conductivity, such as solid electrolytes used in lithium batteries, grain boundary resistance is a significant issue. In such systems, grain boundary diffusion must be measured using approaches different from the conventional Fisher model. Kuwata et al. developed a method to evaluate grain boundary diffusion via cryo-SIMS to obtain high-resolution isotope mapping [401]. In this study, they performed ^6^Li isotope exchange on polycrystalline Li_0.29_La_0.57_TiO_3_ (LLTO) samples, which have a bulk conductivity of 10^−3^ S cm^−1^ at room temperature. Figure 11a illustrates the results of ^6^Li isotope imaging using cryo-SIMS. During cryo-SIMS, the temperature was maintained at − 110°C to quench the Li diffusion. The SIMS image in Figure 11a reveals that the relative ^6^Li fraction, *C*, changes rapidly at the grain boundaries; thus, Li diffusion in the LLTO polycrystals is rate-limiting at these boundaries. Figure 11d shows the profiles of the *C* values along the Black line depicted in the SIMS images, indicating that *C* changes abruptly at the grain boundary. The continuity of the diffusion flux across the interface between the bulk and grain boundaries is expressed as:

(10) $$\begin{array}{c} -D_{\mathrm{bulk}}{\left.\frac{\partial C}{\mathrm{\partial x}}\right|}_{\mathrm{bulk}}=-D_{\mathrm{gb}}\frac{\Delta C_{\mathrm{gb}}}{\delta }, \end{array}$$

Where *∂C*/*∂y*|_bulk_ is the ^6^Li concentration gradient in the bulk nearby the boundary, *Δ*C_gb_ is the difference in the ^6^Li concentration at the grain boundary (Figure 11b). Based on the *D*_bulk_ value obtained from PFG-NMR [408] and the ^6^Li distribution measured by SIMS, the *D*_gb_ was determined to be 7.6 × 10^−14^ cm^2^s^−1^. This technique is applicable for the quantitative evaluation of various interfacial transport processes as rate-limiting steps in batteries.

## 5. Operando TOF-SIMS

**Figure 12.**
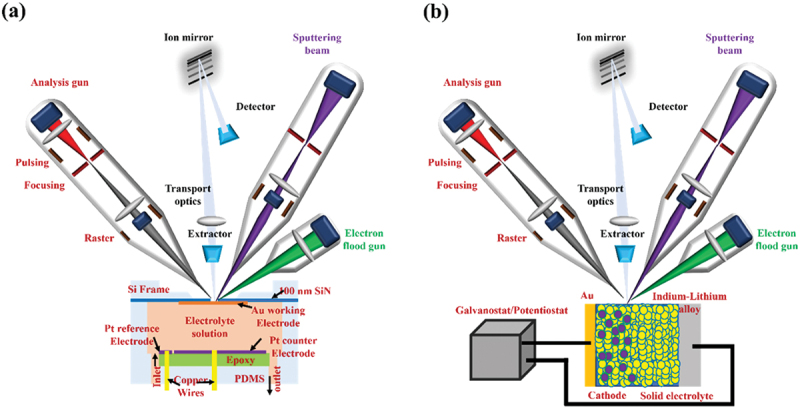
(a) Schematic illustration of the microfluidic device and (b) SSB for real-time and in situ TOF-SIMS analysis of the EEI(Inspired by reference [409,410]).

Operando TOF-SIMS provides real-time insights into dynamic processes, such as ion transport, SEI formation, and interphase evolution, highlighting the effects of electrolyte composition and cycling conditions [53,409–417]. Jusheng Lu et al. reviewed advancements in real-time and in situ analysis, emphasizing the role of SIMS [411]. SIMS employs a focused primary ion beam to ionize sample surfaces, generating secondary ions for mass analysis. While TOF-SIMS has been widely used to study compositional changes at electrode-electrolyte interphase (EEI), its requirement for ultra-high vacuum and dried samples limits in situ and real-time monitoring. To address this, Yu and colleagues developed a vacuum-compatible microfluidic device that integrates a three-electrode configuration, enabling simultaneous electrochemical reactions and TOF-SIMS detection [409,411]. This setup allows real-time observation of potential-dependent changes in key species at the EEI by drilling a micro hole on a SiN membrane and maintaining liquid stability through surface tension, as shown in Figure 12a.

The device facilitated studies of LIB, revealing ion migration, solvent layer formation, and Li salt accumulation during charge-discharge cycles. These findings demonstrated the potential of TOF-SIMS for in situ characterization of dynamic processes at solid-liquid interfaces. Compared to other electrochemical mass spectrometry techniques, TOF-SIMS offers high spatial resolution, rapid sampling, and direct visualization of interfacial evolution, making it invaluable for mechanistic studies of electrochemical reactions. Adding to this, Hai-Lun XIA et.al employed a novel high-vacuum microfluidic electrochemical cell coupled with TOF-SIMS to investigate the redox reaction of coenzyme Q_0_ (CoQ_0_) on gold electrodes [412]. The system facilitated in-situ monitoring of molecular species at the electrode-electrolyte interface under ultra-high vacuum conditions (10^−5^ Pa), revealing detailed dynamics of intermediates such as CoQ_0_H and CoQ_0_H_2_. The findings provide molecular-level insights into the redox mechanism and underscore the potential of TOF-SIMS for advancing the understanding of interfacial electrochemical processes, offering a pathway for broader applications in energy storage and conversion systems. While In situ TOF-SIMS has also provided groundbreaking insights into ion transport through polymeric nanochannels, demonstrating that partial dehydration enables ions to traverse sub-nanometre pores [413]. Chenghai Lu et.al revealed that steric constraints and viscous effects, particularly interactions with functional carboxyl groups, play pivotal roles in shaping hydration dynamics and ion mobility [413]. By reducing the hydration number, ions such as sodium transition to smaller hydrates, facilitating efficient transport. These findings offer critical implications for designing advanced nanofiltration membranes, highlighting TOF-SIMS’s potential to refine our understanding of nano-confined ion behaviour in applications ranging from desalination to battery technologies.

**Figure 13.**
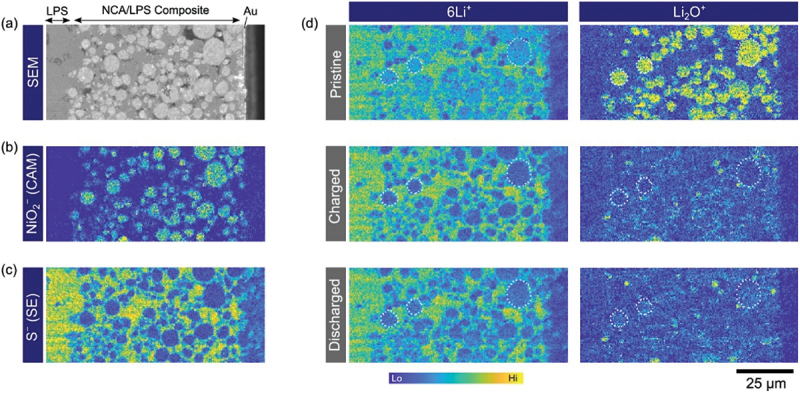
The sem micrograph (a) shows the cross-section of the SSB cell, highlighting key features. Panels (b) and (c) illustrate the local distribution of NiO_2_^−^ and S^−^ fragments, respectively. Panel (d) tracks the evolution of ^6^Li^+^ and Li_2_O^+^ fragment distributions throughout a charge-discharge cycle. White dotted circles indicate the positions of representative NCA particles. (reprinted from ref. [410]; copyright (2021) by the American physical Society).

On top of that Yuji Yamagishi et.al utilizes operando TOF-SIMS to investigate lithium distribution and interfacial degradation in composite cathodes of SSBs [410]. Samples for operando-SIMS were prepared and mounted in an argon-filled glovebox to avoid air exposure, ensuring accurate analysis during battery operation. TOF-SIMS revealed the distribution of lithium (^6^Li^+^ and Li_2_O^+^ fragment) across NCA and LPS particles within the composite electrode, highlighting variations in lithium concentration and confirming lithium extraction during charging and partial reinsertion during discharging. However, ^6^Li^+^ intensity did not recover to initial levels, attributed to Coulombic inefficiency and surface damage from ion beam sputtering, as shown in Figure 13. Selective mapping of Li_2_O^+^ fragments further identified inactive NCA particles due to poor electron and lithium pathways, underscoring the need for optimized electrode microstructures to minimize inactive cathode materials. Moreover, under high-voltage stress, TOF-SIMS detected significant degradation at the NCA/LPS interface. Operando measurements captured the progressive increase of PO_*x*_^−^ and SO_*x*_^−^ fragments during charging and degradation, revealing the formation of compounds such as Li_3_PO_4_ and elemental sulphur [410]. These interfacial reactions are energetically favourable and driven by the higher bond energy of P–O compared to P–S bonds. Notably, the dynamic evolution of these interfacial components during cycling was unique to operando TOF-SIMS, providing real-time insights into the degradation mechanisms of composite electrodes in SSBs [410]. These observations highlight the impact of interfacial instability on battery performance and underscore the need for optimized electrode designs and stable SEI formulations to improve the efficiency and cycle life of SSBs.

**Figure 14.**
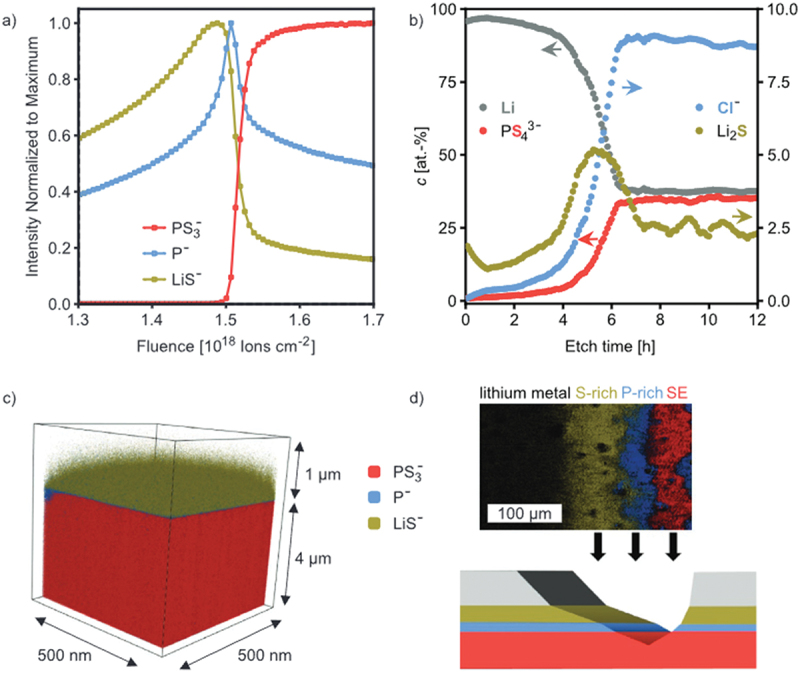
The study investigates the Li/LPSCl interface using TOF-SIMS and XPS techniques. For the analysis, a thin 1 µm layer of lithium was vapor-deposited onto an LPSCl pellet. Key findings include: (a) TOF-SIMS depth profiling provided detailed insights into the elemental distribution across the interface, revealing distinct interfacial gradients. (b) XPS depth profiling quantified atomic percentages of key elements (highlighted in bold and colour), with distinct scales used for clarity. Corresponding arrows and oriented labels indicate the scales associated with the respective data. (c) A 3D representation of the TOF-SIMS depth profile showcased the spatial distribution of elements at the interface. (d) TOF-SIMS imaging of a wedge crater further visualized the interfacial morphology and elemental variations. This multi-faceted approach highlights the complex interfacial chemistry and contributes to the understanding of lithium dynamics in SSBs (reprinted from ref. [53]; copyright (2022) by the John Wiley and sons).

Later the study by Svenja-K. Otto et.al classified SEs based on their interaction with lithium, identifying stable interfaces, SEIs, and mixed-conducting interphases (MCIs) [53]. TOF-SIMS depth profiling provides valuable insights into the three-dimensional structure of interphases in Li/solid electrolyte (SE) systems, especially for thiophosphate SEs like LPSCl, where transmission electron microscopy (TEM) is challenging due to electron beam sensitivity. For instance, TOF-SIMS depth profiles of LPSCl with a 1 µm vapor-deposited lithium layer (Figure 14) reveal the layered nature of the interphase. The use of a linear intensity scale rather than a logarithmic one enhances the clarity of signal variations, such as the sequential maxima for LiS^−^, P^−^, and PS_3_^−^ signals, indicating distinct layers within the interphase. The 3D representation of depth profiles (Figure 14c) confirms this structure, showing an S^−^rich layer near the lithium metal interface, followed by a P-enriched layer closer to the SE interface. Homogeneous lateral distribution of LiS^−^ and P^−^ intensities suggest well-formed, continuous layers. Additionally, wedge-shaped crater imaging (Figure 14d), where sputter dose increases laterally, supports these observations by showing sequential signal intensities such that observation of first LiS^−^, then P^−^, followed by PS_3_^−^, which is consistent with the depth profile. To address the semiquantitative nature of SIMS, XPS depth profiling was also performed. XPS confirms the presence of an S-rich layer (Li_2_S) at lower etch times, preceding the full intensity of substrate signals (PS_4_^3−^). However, compounds like Li_3_P and LiCl were not detected as distinct maxima, possibly due to low concentrations or subtle chemical shifts. These findings align with the TOF-SIMS results, which indicate enrichment of specific elements in different layers, suggesting a layered interphase between the Li_2_S-rich layer and the SE pellet.

For SEIs, such as those on LPSCl, TOF-SIMS revealed layered structures with Li_2_S-rich regions and phosphorus-enriched substrates, with thicknesses of approximately 250 nm. These interphases, critical for limiting degradation, were influenced by deposition methods, with vapor deposition forming smoother layers compared to electrochemical plating. Operando XPS data from Davis et al. [416] further support this interpretation. They observed that Li_2_S formation during lithium plating on LPSCl extends deeper into the SE compared to other reaction products like P and Cl, highlighting the significant role of Li_2_S in the interphase structure. These combined insights underscore the importance of TOF-SIMS and XPS in characterizing layered interphases in SSBs. Adding to this, Qiang Lv et al. investigate high-voltage solid-state lithium metal batteries (SSLMBs) by developing a novel in-situ polymerized solid polymer electrolyte (SPE) with dual-reinforced interfaces to tackle challenges such as interface impedance, dendrite formation, and performance degradation [417]. TOF-SIMS analysis revealed that TMS selectively adsorbs on the cathode, forming a stable CEI enriched with Li_2_SO_4_ and LiF, which resists decomposition under high voltage. Simultaneously, TFEC preferentially adsorbs on the anode, decomposing into LiF and polymeric substances to construct a robust SEI that effectively suppresses dendrite growth. Enhanced ionic conductivity, measured at 6.3 × 10^−4^ S cm^−1^ at room temperature, was attributed to the formation of coordination compounds like [Li(TMS)_*n*_][TFSI], which facilitated efficient Li^+^ dissociation and migration. Depth profiling further demonstrated that the dual-reinforced interfaces reduced interfacial impedance and promoted uniform lithium deposition, supported by smoother Li^+^ distribution across SEI and CEI layers. TOF-SIMS and XPS analyses confirmed the creation of a uniform, LiF-rich SEI layer, while suppressing the formation of organic byproducts that typically impede ionic transport, ensuring stable and efficient performance.

**Figure 15.**
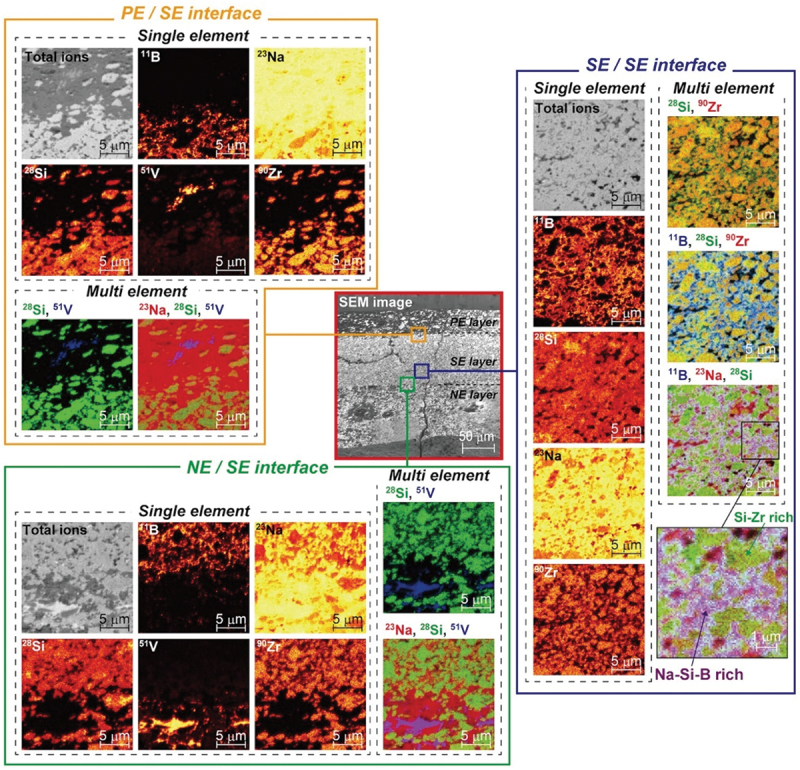
The sem image depicts the observation area with an aspect ratio of 200 μm × 200 μm (highlighted by the red rectangle in the center) alongside TOF-SIMS elemental mapping images of specific interfaces, including PE/SE (orange rectangle, left), SE/SE (blue rectangle, right), and NE/SE (green rectangle, lower), each with an aspect ratio of 20 μm × 20 μm. These mapping images, acquired in positive ion mode, display the distributions of individual elements such as ^11^B, ^23^Na, ^28^Si, ^51^V, and ^90^Zr, as well as overlaid multi-element images that provide a comprehensive view of elemental interactions within the respective interfaces. (reprinted from ref. [415]; copyright (2024) by the John Wiley and sons).

Subsequently, Koji Hiraoka et al. conduct a multi-scale analysis using Operando SEM-EDS, Raman spectroscopy, and TOF-SIMS to investigate charge/discharge mechanisms in oxide-type SSBs with Na_3_V_2_(PO_4_)_3_ (NVP) as both cathode and anode material and Na_3_Zr_2_Si_2_PO_12_ (NZSP) as the solid electrolyte [415]. TOF-SIMS analysis, performed after three cycles in CV measurements, provided detailed elemental distribution mapping across the PE/SE (positive electrode/solid electrolyte), NE/SE (negative electrode/solid electrolyte), and SE/SE interfaces. Sodium (Na) was uniformly distributed in the active layers, but higher concentrations of Na, Si, and B at grain boundaries indicated the formation of ionic conduction pathways via Na – Si – B compounds. At the SE/SE interface, TOF-SIMS detected ^11^B diffusion from adhesive B_2_O_3_ into the grain boundaries (Figure 15), forming Na^+^ -conductive glass-like compounds such as Na_2_O – SiO_2_–B_2_O_3_, which enhanced ionic conductivity (~10^−7^ S cm^−1^ at elevated temperatures) and supported Na migration. TOF-SIMS also revealed Na redistribution during charge/discharge cycles, consistent with redox processes in the NVP active material, and confirmed that Na concentrations in the PE and NE layers changed dynamically during intercalation/deintercalation. Despite cycling, the PE/SE and NE/SE interfaces retained structural integrity, with minimal elemental intermixing. Uniform distributions of Na^+^ and host materials (Si, Zr, V) supported the stability and efficiency of the interface, while TOF-SIMS’s nanometre-scale spatial resolution validated the mechanism of charge/discharge reactions and highlighted the interplay of materials at the micro- and nanoscales.

## 6. Recent advances through machine learning (ML)

This section describes advancements in using TOF-SIMS and machine learning (ML) for analysing battery electrode microstructures and interphases followed by discussing the future perspectives. It highlights the integration of high-resolution 2D SIMS imaging with the SliceGAN algorithm to create realistic 3D microstructures. These models reveal spatial distributions of SEI/CEI interphases and their impact on electrochemical performance, aiding battery optimization. Teo Lombardo et al. present an innovative approach that combines TOF-SIMS with ML to analyse the microstructure and interphase distribution in LIBs electrodes [418]. This methodology focuses on mapping the spatial distribution of active materials, additives, and degradation products, such as the SEI and CEI. In this study, SliceGAN was applied for the first time to secondary ion-based segmented 2D microstructures, with the ultimate goal of generating statistically representative 3D reconstructions of electrode microstructures, incorporating the spatial distribution of primary phases and interphases, as illustrated in Figure 15.

**Figure 16.**
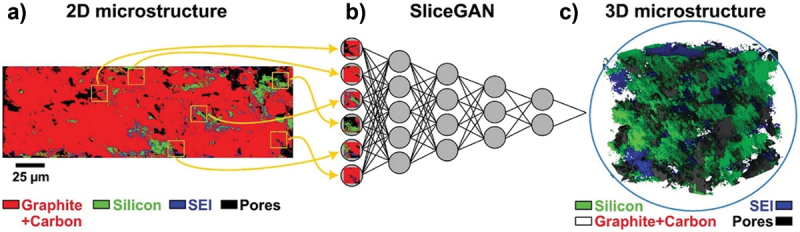
The schematics illustrate the workflow of combining a) SIMS-based 2D electrode microstructure imaging with b) SliceGAN to generate c) statistically reconstructed 3D electrode microstructures that incorporate the spatial distribution of primary phases and interphases. The 3D microstructure (dimensions: 50 × 50 × 50 µm^3^) features the graphite + carbon phase depicted as transparent, allowing a clearer view of the internal structure. SliceGAN, like any generative adversarial network (GAN), comprises two neural networks, the generator and the discriminator though for simplicity, it is represented schematically here as a single network. This visualization highlights the process of translating high-resolution 2D data into a comprehensive 3D model that retains detailed spatial information. (reprinted from ref. [418]; copyright (2023) by the John Wiley and sons).

SliceGAN works by randomly selecting sub-regions from the original 2D images (highlighted in yellow squares in Figure 16) to train two neural networks: the generator, which creates microstructures mimicking the statistical patterns of the original, and the discriminator, which identifies differences between real and artificial images. Training continues until the generator produces 3D microstructures indistinguishable from the original 2D microstructures, successfully extending its learning into three-dimensional space (Figure 16C). This approach, combining high-resolution SIMS imaging with SliceGAN, currently stands as the only method capable of producing large-scale, realistic 3D microstructures that include detailed spatial distributions of SEI and CEI interphases. These microstructures can be integrated into 3D electrochemical models to explore the impact of SEI/CEI spatial distributions on electrochemical performance, an area previously limited to stochastic modelling. While implementing such highly resolved microstructures into 3D models remains computationally challenging, this work provides a valuable starting point for other researchers to refine electrochemical models to incorporate interphase distributions. The 2D SIMS-based microstructures developed in this study are published alongside the article, offering the battery research community access to these datasets for future modelling efforts. The combination of TOF-SIMS and SliceGAN represents a significant advancement in battery electrode microstructure characterization, paving the way for new insights into the relationships between microstructures, interphases, and battery performance.

## 7. Future perspective of TOF-SIMS

**Figure 17.**
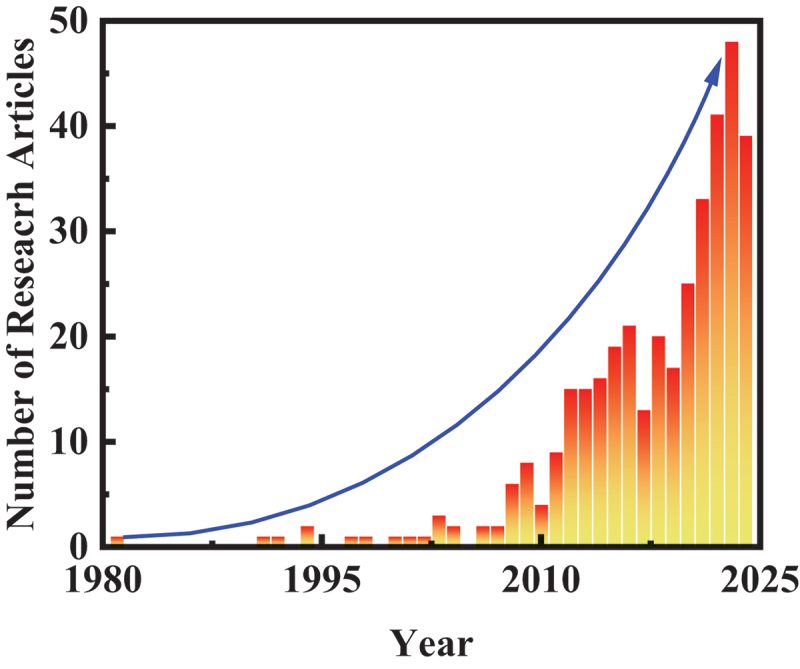
Exponential growth in the use of TOF-SIMS in battery research from 1980 to 2024. The figure illustrates the increasing number of publications and studies leveraging TOF-SIMS for battery-related investigations, highlighting its growing importance due to its high sensitivity, spatial resolution, and capability to provide detailed chemical analysis of interfaces and bulk materials. This trend reflects the expanding role of TOF-SIMS in advancing the understanding and optimization of modern battery systems.

Overall, this review highlights the significant role of TOF-SIMS in advancing battery research while identifying several promising avenues for future exploration. Over the years, from 1980 to early 2025, there has been exponential growth in the application of SIMS in battery research, as illustrated in Figure 17. This surge can be attributed to the well-known advantages of TOF-SIMS, such as its high sensitivity, spatial resolution, and ability to provide detailed chemical mapping at interfaces and in bulk materials. These capabilities, combined with its unmatched ability to perform high-resolution depth profiling and isotopic labelling, have made TOF-SIMS indispensable for characterizing dynamic processes like SEI and CEI evolution, lithium-ion migration, and additive interactions. Future research is dignified to leverage TOF-SIMS for real-time analysis of interfacial dynamics under operational conditions, such as operando studies, and its integration with complementary techniques like XPS, TEM, SEM, NMR, and Raman spectroscopy promises to bridge gaps in surface and bulk characterization, enabling a holistic analysis of complex systems [51,71,411,419–423].

The future of ToF-SIMS in battery research is closely tied to continued improvements in instrumentation. Its ability to generate high-resolution, three-dimensional chemical maps provides unparalleled insights into interphases, grain boundaries, and buried interfaces that govern electrochemical performance [58,424–434]. The development of operando and in situ TOF-SIMS methods, including vacuum-compatible electrochemical cells and cryogenic adaptations, enables real-time monitoring of interfacial reactions and transient processes such as dendrite initiation, interphase growth, and redox decomposition. Equally transformative is the integration of TOF-SIMS with machine learning and multivariate statistical models, which facilitates automated interpretation of complex spectra, realistic 3D reconstruction of electrode architectures, and predictive evaluation of degradation pathways. Such advancements will accelerate the rational design of interfacial chemistries and microstructures for safer and longer-lasting batteries. Beyond lithium-ion technology, TOF-SIMS is expected to expand its application space to emerging systems such as lithium – sulfur, lithium – air, sodium-ion, potassium-ion, and multivalent (Mg, Zn, Ca) batteries, where interfacial instability remains a major bottleneck. The technique’s sensitivity to light elements and molecular fragments allows for direct visualization of sulfur species migration, interfacial decomposition, and multi-ion transport. Isotope labeling combined with depth profiling further provides quantitative diffusion coefficients, revealing ion migration pathways across complex electrolytes and electrode composites. These developments position TOF-SIMS not only as a diagnostic tool but also as a predictive platform that bridges nanoscale characterization with electrochemical modelling, ultimately guiding the optimization of materials and interfaces for emerging energy storage technologies. Moreover, as datasets generated by ToF-SIMS become increasingly large and complex (as discussed above), enhanced data processing techniques will be essential. Artificial intelligence (AI) and ML offer significant potential to transform ToF-SIMS analysis [58,424–434]. By recognizing spectral patterns, differentiating chemically similar species, and deconvoluting overlapping peaks, AI-assisted analysis will accelerate interpretation and reduce operator bias. Machine learning models can also predict chemical changes in SEI and CEI layers, facilitating the identification of previously unobserved interfacial phenomena. Furthermore, the integration of statistical and multivariate analysis with high-throughput ToF-SIMS datasets will allow systematic correlation of interfacial chemistry with electrochemical performance, guiding the rational design of next-generation battery materials. As a result, TOF-SIMS is poised to be a cornerstone of next-generation energy storage research, unlocking new possibilities for high-performance, sustainable, and safe battery technologies.

Despite its significant advantages, TOF-SIMS is constrained by several fundamental limitations that complicate its application in battery research. The requirement for ultra-high vacuum conditions restricts the direct analysis of liquid electrolytes and dynamic interfaces under realistic operating conditions. Although adaptations such as cryo-TIMS and microfluidic-compatible environments have mitigated these challenges, their implementation remains technically demanding and limited in scope. Additionally, TOF-SIMS is inherently semi-quantitative, as ionization yields strongly depend on the chemical matrix, leading to signal intensities that cannot be directly converted into absolute concentrations. Calibration with standards and normalization procedures improve data reliability, but accurate quantification across diverse chemistries remains an unresolved challenge. Other drawbacks include potential beam-induced damage and knock-on effects during sputtering, which may distort delicate interphase structures such as the SEI and CEI. Cluster ion beams and low-energy sputtering reduce these artifacts but do not eliminate them entirely. Moreover, the multidimensional datasets generated by TOF-SIMS, encompassing spatial, depth, and spectral domains – require sophisticated data analysis pipelines, and misinterpretation remains a risk in the absence of advanced computational frameworks. Finally, the technique is time- and cost-intensive compared to complementary methods such as XPS, Raman, or NMR, limiting its widespread adoption in high-throughput industrial screening. Overcoming these limitations will be critical to fully realizing the potential of TOF-SIMS in both fundamental studies and applied battery diagnostics.
